# Suppression of trinucleotide repeat expansion in spermatogenic cells in Huntington’s disease

**DOI:** 10.1007/s10815-022-02594-x

**Published:** 2022-09-06

**Authors:** In K. Cho, Charles A. Easley, Anthony W. S. Chan

**Affiliations:** 1 Department of Human Genetics, Emory University School of Medicine, Atlanta, GA USA; 2 Division of Neuropharmacology and Neurologic Diseases, Emory National Primate Research Center, Emory University, Atlanta, GA USA; 3 Department of Environmental Health Sciences, College of Public Health, University of Georgia, Athens, GA USA; 4 Regenerative Bioscience Center, University of Georgia, Athens, GA USA; 5 Environmental Health Science and Regenerative Bioscience Center, College of Public Health, University of Georgia, Edgar L. Rhodes Center for Animal and Dairy Science RM 432, 425 River Rd, Athens, GA 30602 USA; 6 Present Address: Center of Scientific Review (CSR), National Institutes of Health, Bethesda, USA

**Keywords:** Huntington’s disease, Trinucleotide repeats, Spermatogenesis, Drug discovery, Cytarabine (AraC), Aspirin

## Abstract

Trinucleotide repeats (TNRs) are dispersed throughout the human genome. About 20 loci are related to human diseases, such as Huntington’s disease (HD). A larger TNR instability is predominantly observed in the paternal germ cells in some TNR disorders. Suppressing the expansion during spermatogenesis can provide a unique opportunity to end the vicious cycle of genetic anticipation. Here, using an in vitro differentiation method to derive advanced spermatogenic cells, we investigated the efficacy of two therapeutic agents, araC (cytarabine) and aspirin, on stabilizing TNRs in spermatogenic cells. Two WT patient-derived induced pluripotent stem cell (iPSC) lines and two HD hiPSC lines, with 44 Q and 180 Q, were differentiated into spermatogonial stem cell-like cells (SSCLCs). Both HD cell lines showed CAG tract expansion in SSCLC. When treated with araC and aspirin, HD1 showed moderate but not statistically significant stabilization of TNR. In HD2, 10 nM of aspirin and araC showed significant stabilization of TNR. All cell lines showed increased DNA damage response (DDR) gene expression in SSCLCs while more genes were significantly induced in HD SSCLC. In HD1, araC and aspirin treatment showed general suppression of DNA damage response genes. In HD2, only *FAN1*, *OGG1*, and *PCNA* showed significant suppression. When the methylation profile of HD cells was analyzed, *FAN1* and *OGG1* showed significant hypermethylation after the aspirin and araC treatment in SSCLC compared to the control. This study underscores the utility of our in vitro spermatogenesis model to study and develop therapies for TNR disorders such as HD.

**Supplementary Information:**

The online version contains supplementary material available at 10.1007/s10815-022-02594-x.

## Introduction

Trinucleotide repeats (TNRs) are dispersed throughout the human genome, and about 20 loci are related to human diseases, such as Huntington’s disease (HD), fragile X syndrome (FXS), and spinocerebellar ataxia (SCA) [1–3]. HD is a monogenic neurodegenerative disease characterized by progressive brain atrophy in the striatum, cortex, and other brain regions. Individuals with more than 35 CAG repeats (polyglutamine repeats; polyQ) in exon 1 of the huntingtin (*HTT*) gene will develop HD [4]. Furthermore, the age of onset is inversely correlated to the size of the CAG repeat. TNRs are unstable and are prone to changes (i.e., expansions and contractions). Although peripheral tissues and central nervous tissues, such as the brain, show TNR expansions in the various animal models and human patients [5–7], a larger TNR instability is predominantly observed in the paternal germ cells in TNR disorders, including HD and SCA. The expansion in germ cells results in genetic anticipation where paternal progeny gets expanded TNR [8–11]. Male spermatogenesis is a multistep process involving DNA replication, ligation, proof-reading, and repair, providing ample opportunities for errors, such as CAG expansion. However, it is still unknown whether these CAG repeats can be stabilized to reduce the expansion in humans with small molecules. TNR expansion in rodents occurs in the post-meiotic stage [12], while expansions occur in pre-meiotic and post-meiotic sperm cells in humans [13]. The main factor influencing TNR instability is the number of repeats, which makes HD patients with larger number of polyQ repeats more vulnerable to environmental insult and susceptible to changes [14].

With the recent development of stem cell technologies, various cell types can be derived to investigate the underlying mechanism of diseases in a dish. Our team has developed and utilized in vitro directed differentiation method to derive advanced spermatogenic cells using the protocol based on our recent success in deriving haploid spermatids from human PSCs. In recent developments, directed in vitro differentiation of deriving spermatogenic cells have improved [15–17] and used to access developmental toxicity and environmental toxicants [18–21]. However, among the “gold standards” in evaluating in vitro-derived germ cells, the goal for demonstrating the faithful meiosis is the ability to fertilize oocytes and produce embryos [22]. In a recently published article, we have demonstrated that upon intracytoplasmic sperm injection, rhesus oocytes were successfully fertilized by the in vitro differentiated spermatids, and some developed into blastocytes [23]. Also, we have demonstrated that non-human primate (NHP) embryonic stem cell (nhpESC) derived spermatogonial stem cell-like cells (SSCLCs) can replicate the CAG repeat expansion in vitro [24]. We have demonstrated that the daily expansion rate of SSCLC was more significant than the expansion rates of the extended culture of nhpESC [24]. Suppression of expansion or even inducing contraction of TNR in paternal germline will provide an opportunity to eradicate the devastating effect of genetic anticipation in TNR disorders. Recent reports have suggested that TNR instabilities can be promoted by environmental stresses [25], hormonal disruption [26], and chemical exposures [27]. Gomes-Pereira and Monckton showed chronic exposure to chemical treatment could alter CAG*CTG repeat size in *Dmt*-D myotonic dystrophy mouse cell lines [27]. Of eight chemicals tested, araC and aspirin resulted in large contractions, and caffeine showed the largest expansion [27]. Therefore, we selected araC and aspirin as contracting candidates and caffeine as an expansion control.

Here, we utilized human induced pluripotent stem cell (hiPSC) to investigate the efficacy of two therapeutic agents, araC (cytarabine) and aspirin, on stabilizing TNR in spermatogenic cells using our in vitro SSCLC directed differentiation in HD. The differentiation process resulted in expanded CAG repeats in spermatogenic cells. Moreover, treating cells during the differentiation process with subclinical dosages of araC and aspirin stabilized CAG expansion in spermatogenic cells. This study demonstrates that this platform can be used to discover new therapeutic targets, provide a method to investigate the mechanisms involved in germline TNR expansion in different cell types, and has further applications in other TNR disorders with the paternal origin of TNR expansion such as dentatorubral-pallidoluysian atrophy (DRPLA), oculopharyngeal muscular dystrophy (OPMD), SCA, spinomuscular bulbar atrophy (SMBA), and myotonic dystrophy (DM).

## Results

**Fig. 1:**
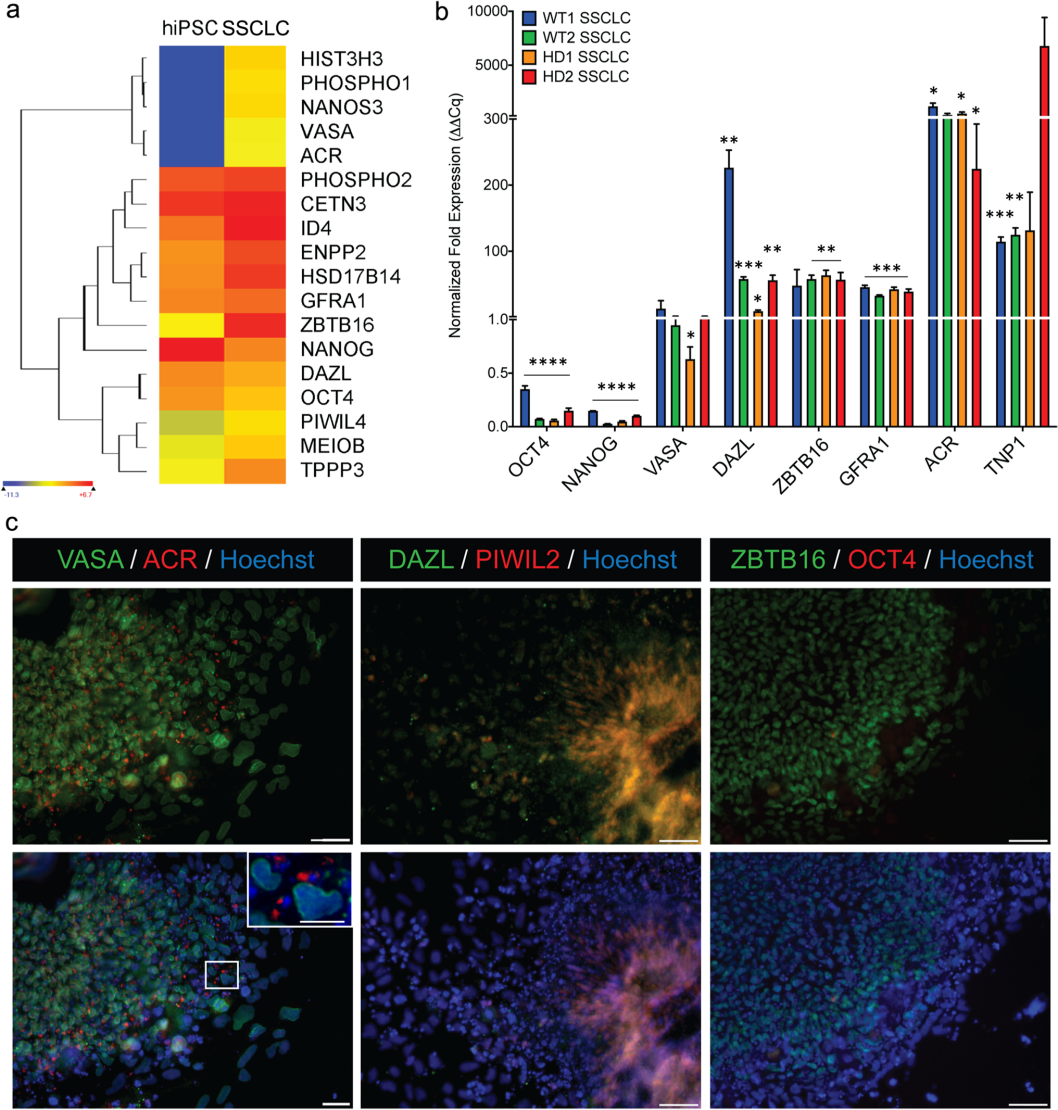
Differentiation confirmation of hiPSC to SSCLC.** A** Raw read counts (RPKM) of hiPSC compared to day 10 sample showing higher expression of some of the spermatogenesis-specific transcripts: *VASA*, *ACR*, *GFRA1*, and *ZBTB16*. **B** Quantitative real-time polymerase chain reaction (qRT-PCR) showing downregulation of pluripotency markers expressions such as *OCT4* and *NANOG* for all cell lines (for all *p* < 0.00005), and upregulation of spermatogonial stem cells like cells markers such as *VASA*, *DAZL*, *ZBTB16*, *GFRA1*, *ACR*, and *TNP1* (*n* = 3). **C** Representative immunofluorescence showing expression of VASA, ACR, DAZL, PIWIL2, and ZBTB16 but no OCT4 (scale = 100 µm, insert scale = 50 µm)

**Fig. 2:**
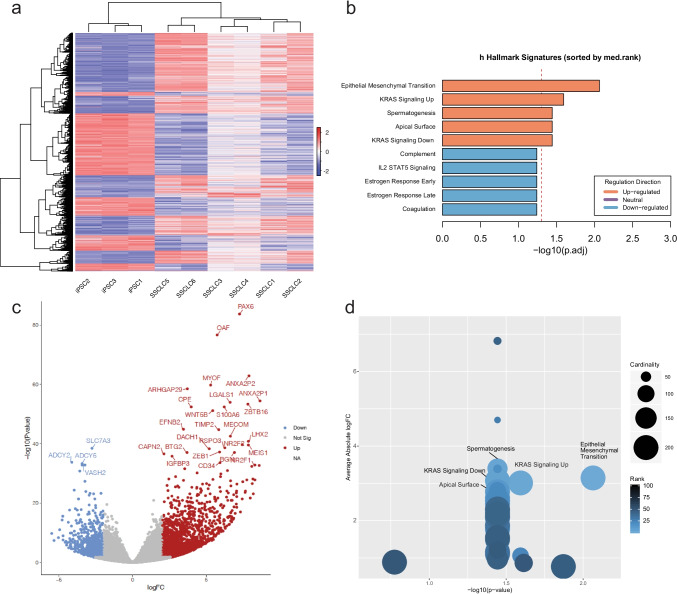
RNA-seq analysis of differentially expressed genes of iPSC and SSCLC.** A** Hierarchical clustering and the sample-to-sample cluster of differentially expressed genes showing clustering of iPSCs and SSCLCs. **B** Gene set enrichment analysis of both upregulated and downregulated genes showing spermatogenesis as one of the top enriched hallmark signatures (22/135, *p* = 0.036) while downregulation of estrogen response hallmark signatures (49/200, *p* = 0.054). **C** Volcano plot of differentially expressed genes showing induced expression of a spermatogenic gene such as *PAX6*, *TIMP2*, *MECOM*, and *ZBTB16*. **D** Gene set enrichment analysis (GSEA) of induced genes after the differentiation shows spermatogenesis as one of the hallmark signatures enriched after the differentiation

Two WT hiPSC cells (BJ-WT1 and ND41658-WT2) and two HD hiPSC cells (ND38547-HD1 with 44 Q and ND36999-HD2 with 180 Q) were differentiated into spermatogonial stem cell-like cells (SSCLCs) in vitro following the established protocol [16] (Fig. 1 and Supplementary Information Fig. 1). Differentiation efficiency of all four cell lines was evaluated by RNA-seq (Fig. 1a, Fig. 2), RT-PCR (Fig. 1b), and immunofluorescence (Fig. 1c, Supplementary Information Fig. 1). When RNA-seq data of day 10 samples (SSCLCs) were compared to iPSC, spermatogenic specific markers such as *VASA*, *ACR*, *GFRA1*, *ZBTB16*, and *MEIOB* were induced in day 10 samples compared to iPSC (Fig. 1a) (*n* = 3 for iPSC and *n* = 6 for SSCLC). Quantitative real-time polymerase chain reaction (qRT-PCR) analysis of selected genes (*OCT4*, *NANOG*, *VASA*, *DAZL*, *ZBTB16*, *GFRA1*, *ACR*, and *TNP1*) showed the significant downregulation of pluripotent stem cell markers (*OCT4* and *NANOG*, *p* < 0.00005 for both) and significant upregulation of spermatogenic specific transcripts (*VASA*, *DAZL*, *ZBTB16*, *GFRA1*, *ACR*, and *TNP1*) (*n* = 3 for each cell lines). The expression of VASA, ZBTB16, PIWIL2, DAZL, and ACR were confirmed with immunofluorescence while no OCT4 expression was detected (Fig. 1c, Supplementary Information Fig. 1). Hierarchical clustering of RNA-sequence analysis of differentially expressed (DE) genes from iPSC and SSCLC showed clustering of iPSCs and SSCLCs (Fig. 2a). Ensemble of Gene Set Enrichment Analyses (EGSEA) of significantly induced genes showed spermatogenesis (M5951) as the third-highest ranked hallmark gene signatures (number of genes = 22/135, *p* = 0.036, average log fold change (FC) = 3.4) (Fig. 2b). A total of 6133 genes were significantly differentially expressed when SSCLCs were compared to iPSCs (Fig. 2a, c). Genes involved in spermatogenesis and specifically associated with testis development were among those significantly induced genes (Fig. 2c).

**Fig. 3:**
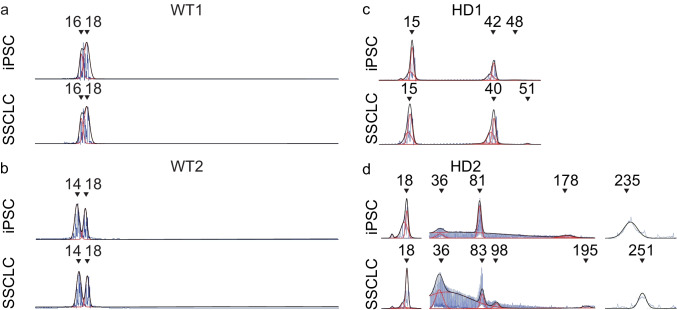
Representative electropherograms of four cell lines used in this study before (iPSC) and after the differentiation (SSCLC). **A**, **B** Both wild-type cell lines did not change CAG size before and after the differentiation process. **C** HD1 showed a slight increase in the more significant allele of the CAG repeat after the differentiation from 48 to 51 Q, while 15 Q did not change. **D** HD2 showed a general increase in CAG repeat sizes for intermediate alleles and larger alleles, while the smallest allele did not change. The intermediate allele size changed from 81 to 83 Q and 98 Q and 178 Q to 195 Q. The larger allele changed from 235 to 251 Q (*Y*-axis represents relative fluorescence intensity, RFU)

**Fig. 4:**
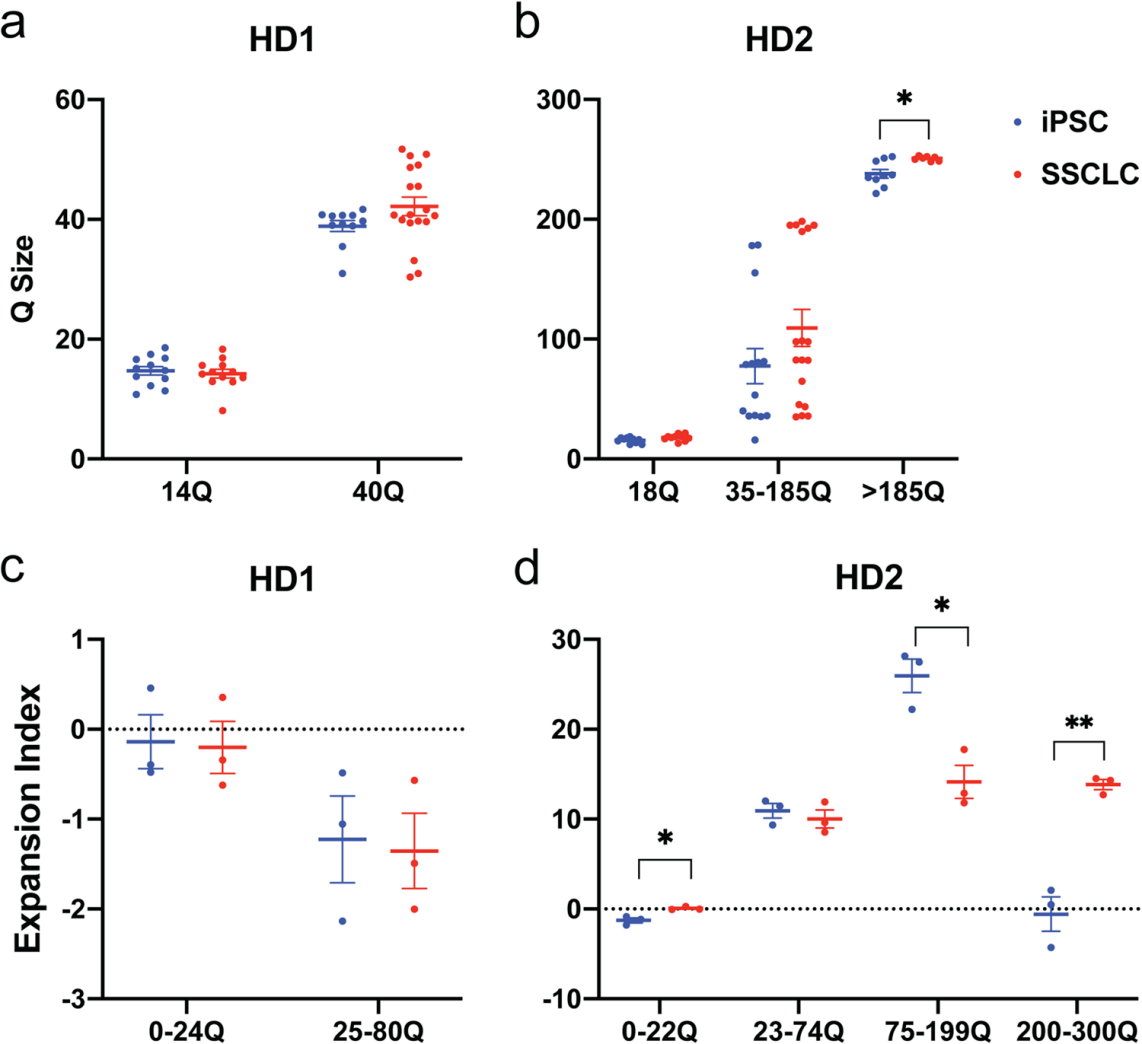
TNR changes during differentiation.** A** Curve fit analysis of HD1 before and after the differentiation showing no significant change for both small allele (*x̄* = 14.72 Q to *x̄* = 14.23 Q, *p* = 0.64) and larger allele (*x̄* = 38.88 Q to *x̄* = 42.17 Q, *p* = 0.132). **B** Curve fit analysis of HD2 showed increase of 2 Q of the 18 Q allele from *x̄* = 15.62 Q to *x̄* = 17.88 Q, which was not statistically significant (*p* = 0.0534). The intermediate allele increased 31.85 Q from *x̄* = 77.54 Q to *x̄* = 109.4 Q but was not significant (*p* = 0.154). The largest allele significantly increased after the differentiation from *x̄* = 238 Q to *x̄* = 250.8 Q (*p* = 0.00838). **C** The expansion index showed no changes of small allele (*p* = 0.885) and large allele (*p* = 0.850) for HD1 after the differentiation. **D** The expansion index showed significant increase of small allele (*p* = 0.0102), decrease in larger intermediate allele (*p* = 0.0107), and the increase in the larger allele (*p* = 0.00193)

To evaluate whether in vitro differentiation process can recapitulate the TNR expansion as in in vivo spermatogenesis, genomic DNA was extracted from iPSC and SSCLC, and the size of TNRs was analyzed. Both wild-type cells (WT1 and WT2) did not change CAG size before and after the differentiation process (Fig. 3a, b, Supplementary Information Fig. 2). However, after the differentiation process, HD2 showed significant expansion in CAG repeat sizes in SSCLC compared to iPSC (Fig. 3d; Fig. 4b, d; Supplementary Information Fig. 3). Although HD1 showed a slight increase in CAG repeat size in large allele, the change was not significant with both curve fit analysis (Fig. 4a, *x̄* = 38.88 Q to *x̄* = 42.17 Q, *p* = 0.132) and expansion index analysis (Fig. 4c, p = 0.850). HD2 showed a significant increase in CAG repeat size after the differentiation (Fig. 4b, d). With curve-fit analysis, HD2 showed significant increase of large allele (Fig. 4b, from *x̄* = 238 Q to *x̄* = 250.8 Q, *p* = 0.00838). Also, expansion index increased significantly for small allele (*p* = 0.0102) and larger allele (*p* = 0.00193).

**Fig. 5:**
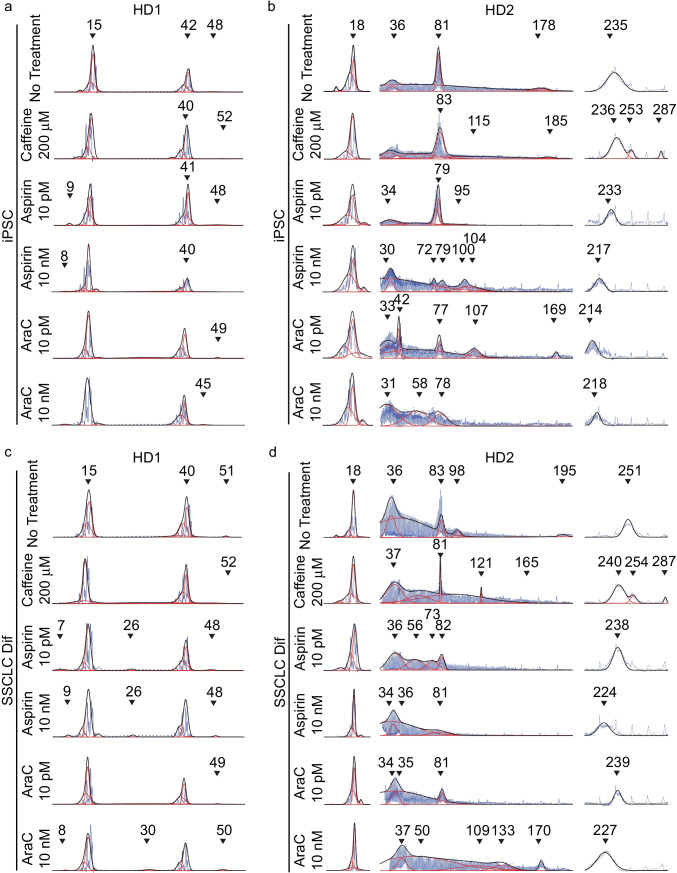
Representative electropherograms of iPSC and SSCLC chemical treated cells. **A** Representative electropherograms showing the impact of caffeine, aspirin, and araC treatment in iPSC. Caffeine slightly increased 48 Q to 52 Q while aspirin 10 nM treatment showed a decrease in 42 Q to 40 Q, and no 48 Q allele was observed. AraC treatment showed a slight decrease in 48 Q to 45 Q. **B** Chemical treatment of HD2 at iPSC state showed a slight increase of larger allele with caffeine treatment from 235 to 236 Q, 253 Q, and 287 Q. Aspirin treatment showed a general decrease in repeat size. In contrast, the higher concentration of aspirin showed a more significant decrease in CAG repeat size. AraC treatment showed a decrease in CAG repeat size, especially for the intermediate allele. At the same time, the large allele also showed a decrease in the repeat size from 235 to 218 Q. **C** Chemical treatment during SSCLC differentiation of HD1 showed a general decrease with aspirin and araC treatment. At the same time, a slight increase in caffeine was observed. **D** HD2 treated with caffeine showed a general increase in CAG repeat size, while aspirin and araC showed a general decrease in CAG repeat size (*Y*-axis represents relative fluorescence intensity, RFU)

**Fig. 6:**
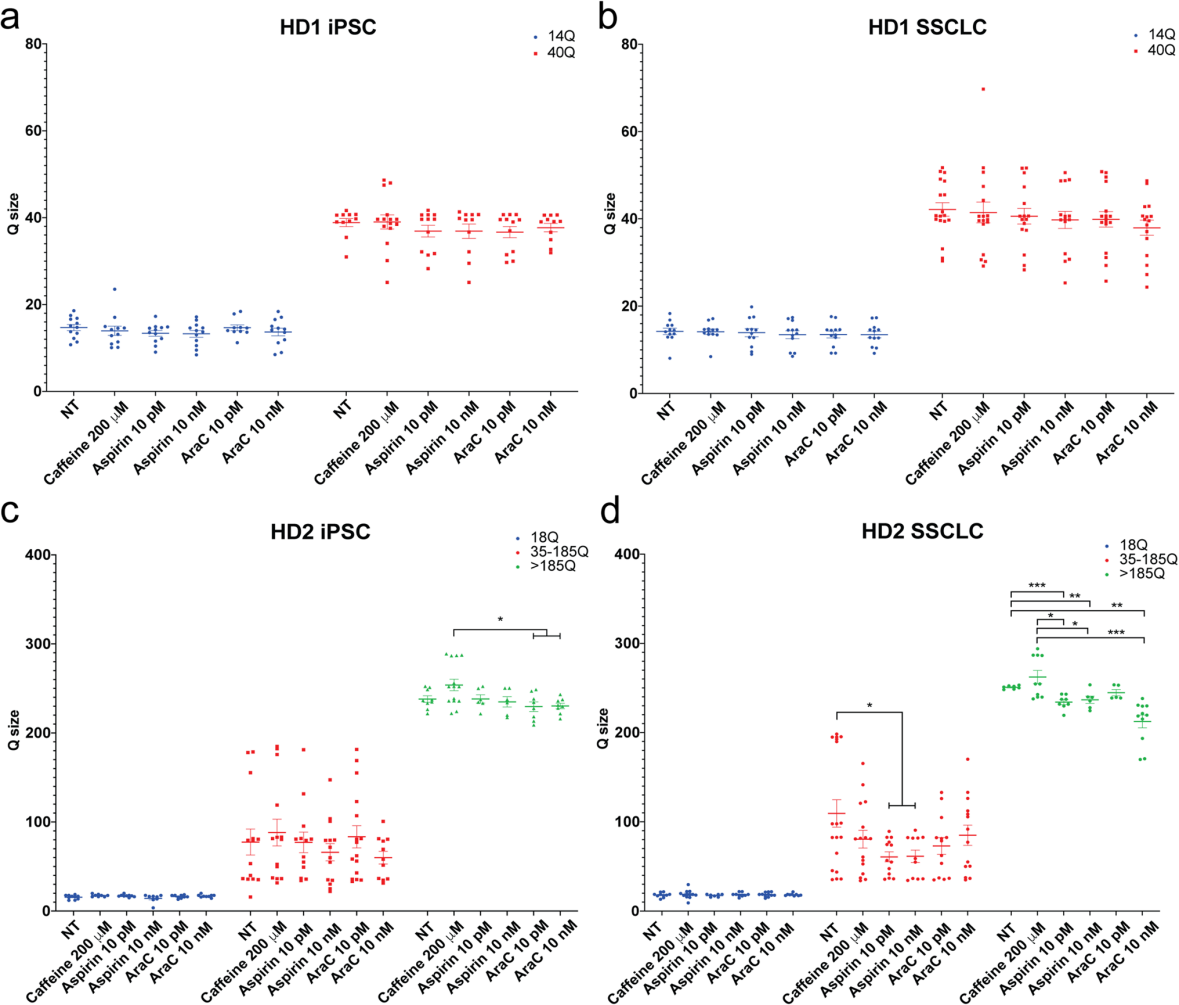
Curve-fit data analysis of repeat sizes.** A** Chemical treatment of HD1 iPSC did not significantly change the CAG repeat size. **B** Although aspirin and araC treatment of HD1 SSCLC generally reduced the CAG repeat size of the larger allele, the changes were not statistically significant. **C** Chemical treatment of HD2 iPSC did not show a significant reduction in CAG repeat size with aspirin and araC treatment. However, when compared to caffeine treated cells, both concentrations of araC 10 pM and 10 nM treated cells showed significantly reduced size of CAG repeat size (*p* = 0.0187 and *p* = 0.0161, respectively). **D** Both concentrations of aspirin treatment (10 pM and 10 nM) during SSCLC differentiation stabilized intermediate allele size when compared to control group (*x̄* = 109.4 Q to *x̄* = 60.74 Q and 61.39 Q, *p* = 0.015 and 0.0273 respectively). Both aspirin and araC significantly stabilized larger alleles compared to the no treatment group. The aspirin treatment showed significant stabilization from *x̄* = 250.8 Q to *x̄* = 234.2 Q or 10 pM and *x̄* = 236.8 Q for 10 nM (*p* < 0.001 and = 0.00454 respectively). SSCLC treated with 10 nM of araC showed significant stabilization of larger allele from *x̄* = 250.8 Q to *x̄* = 212.5 Q (*p* < 0.001) but not for 10 pM treatments (*x̄* = 244.7 Q, *p* = 0.0736). For all groups, the mean and the standard error with individual data points were plotted. Multiple unpaired *t*-tests were conducted to calculate the statistical significance. *n* = 3, *p < 0.05, **p < 0.005, ***p < 0.0005, ****p < 0.00005

Then, we evaluated whether treating cells with chemicals at iPSC state or during SSCLC differentiation can modulate CAG repeat sizes. Both iPSCs and cells undergoing SSCLC differentiation were treated with 200 mM caffeine, 10 pM and 10 nM aspirin, and 10 pM and 10 nM araC for 10 days. Although slight increase of CAG repeat sizes were observed when cells were treated with 200 mM caffeine (Figs. 5 and 6), no statistically significant changes were observed in all cells (Figs. 5 and 6). In HD1, no statistically significant changes were observed with aspirin and araC treatments (Fig. 6, Supplementary Information Fig. 4). HD2 iPSC treated with 10 pM and 10 nM of araC showed a significant decrease in CAG repeat size compared to 200 mM caffeine group (Fig. 6c, p = 0.0187 and *p* = 0.0161 respectively). HD2 SSCLC treated with 10 pM and 10 nM of aspirin showed significant decrease in intermediate allele (Fig. 6d, *x̄* = 109.4 Q to *x̄* = 60.74 Q and 61.39 Q, *p* = 0.015 and 0.0273 respectively). Both aspirin and araC significantly stabilized larger allele compared to no treatment group. The aspirin treatment showed significant stabilization from *x̄* = 250.8 Q to *x̄* = 234.2 Q for 10 pM and *x̄* = 236.8 Q for 10 nM (*p* < 0.001 and 0.00454 respectively). SSCLC treated with 10 nM of araC showed significant stabilization of larger allele from *x̄* = 250.8 Q to *x̄* = 212.5 Q (*p* < 0.001) but not for 10 pM treatments (*x̄* = 244.7 Q, *p* = 0.0736). Based on expansion index analysis, small allele from all HD2 SSCLC showed significantly increased expansion index compared to iPSC (Supplementary Information Fig. 4b). AraC 10 nM treatment of iPSC showed significantly lower expansion index compared to caffeine and aspirin (10 pM and 10 nM) treatment group (Supplementary Information Fig. 4b). Compared to all treatment groups before the differentiation, SSCLC control group showed increased expansion index after the differentiation for the larger allele (Supplementary Information Fig. 4b, 200–300 Q, purple circles). With chemical treatments, both aspirin and araC treatment showed significant decrease in expansion index compared to no treatment control in SSCLC for all concentration groups (Supplementary Information Fig. 4b). Also, higher concentration of araC (10 nM) showed significant decrease in expansion index compared to lower 10 pM concentration of araC (Supplementary Information Fig. 4b, *p* < 0.001). The larger intermediate alleles (75–199 Q) showed significant decrease in expansion index when SSCLC no treatment group was compared to both control and caffeine treatment group (Supplementary Information Fig. 4b). Both aspirin concentration of 10 pM and 10 nM showed reduced expansion index compared to the control (Supplementary Information Fig. 4b). SSCLC treated with 10 nM araC increased expansion index compared to the control (Supplementary Information Fig. 4b) and both concentration of aspirin of 10 pM and 10 nM (Supplementary Information Fig. 4b).

**Fig. 7:**
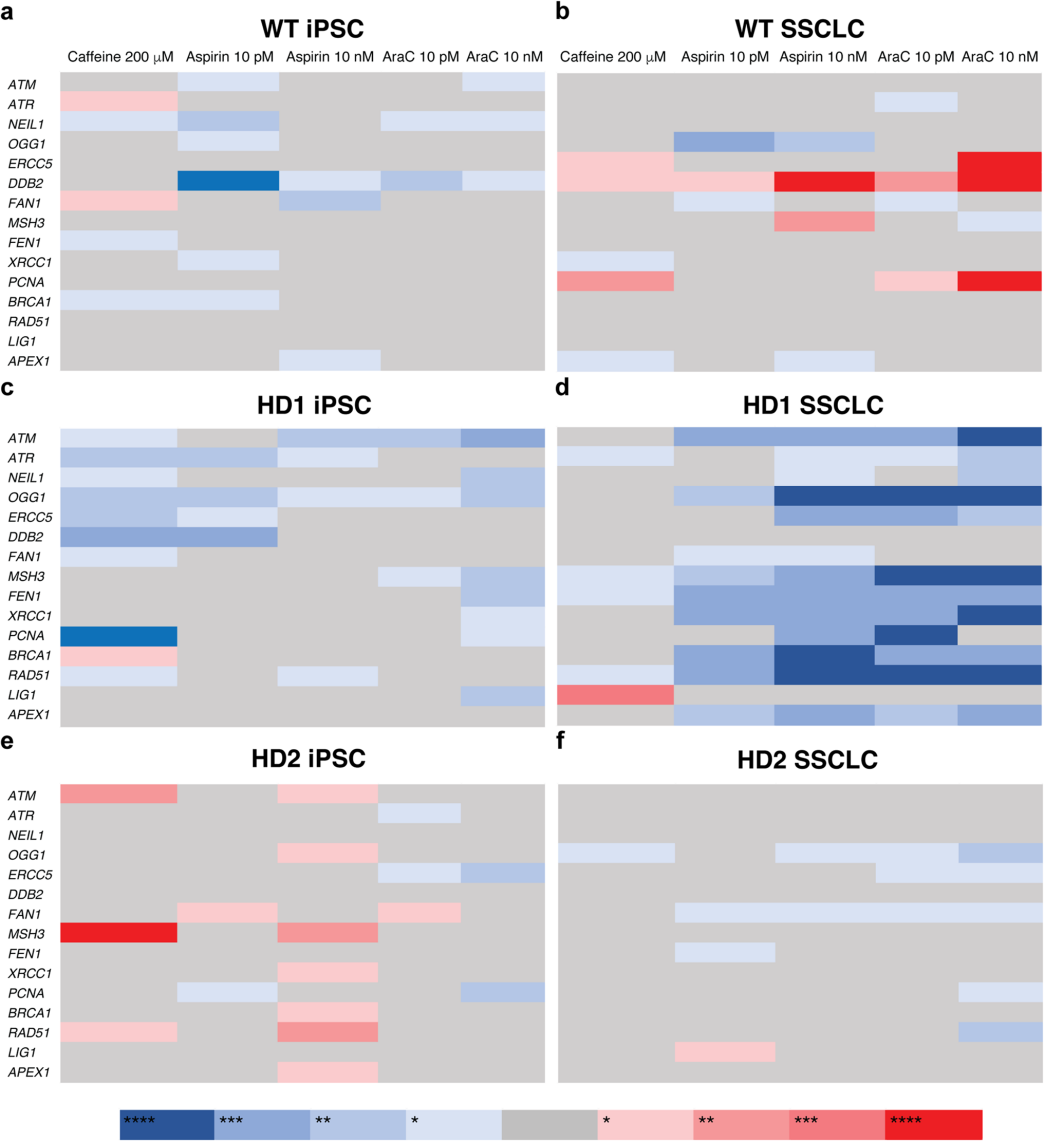
A summary plot of DDR gene expression relative to control. **a** Caffeine treatment induced expression of *ATR* (*p* = 0.0477) and *FAN1* (*p* = 0.00687) while aspirin and araC treatment suppressed *DDB2* expression. **b** Caffeine, aspirin, and araC treatment induced expression of *DDB2* while aspirin suppressed *OGG1* expression, and araC induced *PCNA* expression. **c** In general, caffeine suppresses DDR gene expression except for *BRCA1* (*p* = 0.0052), and aspirin and araC suppressed *OGG1* expression. *ATR* expression was suppressed with aspirin treatment, while *ATM* and *MSH3* were suppressed with araC treatment. **d** Both aspirin and araC treatment suppressed most of DDR genes while caffeine induced expression of *LIG1* in SSCLC (*p* < 0.001). **e** Caffeine and 10 nM of aspirin treatment induced *ATM*, *MSH3*, and *RAD51* in HD2 iPSC, while *ATR*, *ERCC5*, and *PCNA* expressions were suppressed in the cells treated with araC. Also, 10 nM of aspirin induced most of the DDR genes. **f** Except for *OGG1*, most DDR genes did not change expression when treated with caffeine. SSCLC treated with aspirin and araC suppressed *OGG1* and *FAN1* expression, and araC suppressed *ERCC5* while only 10 nM of araC suppressed *PCNA* and *RAD51* expression. Multiple unpaired t-tßests were conducted to calculate the statistical significance (*n* = 6 to 12 based on the sample availability). *p < 0.05, **p < 0.005, ***p < 0.0005, ****p < 0.00005

To investigate mechanisms involved in the changes in CAG repeat size during the differentiation process and with the chemical treatment, the gene expression of a panel of genes involved in the DNA damage response (DDR) pathway was analyzed utilizing RT-PCR (Supplementary Information Figs. 5, 6). At iPSC state, both WT and HD showed similar DDR gene expression (Supplementary Information Fig. 5a). In SSCLC, HD generally showed induced expression of DDR genes (Supplementary Information Figs. 5b, 6a). Since the presence of expanded CAG repeat track in *HTT* might affect the DDR gene expression in SSCLC, the expression of DDR genes were separately compared in WT and HD cells (Supplementary Information Fig. 5c, d). In both WT and HD, generally DDR gene expressions were higher in SSCLC (Supplementary Information Fig. 5c, d). However, *DDB2* and *PCNA* expressions were significantly lower in WT SSCLC than WT iPSC (Supplementary Information Fig. 5c). In normal tissues, the expression of *HTT* is elevated in the brain, skin, and testis [28]. Both WT and HD cells show significant upregulation in *HTT* expression in SSCLC compared to iPSC (Supplementary Information Fig. 7). In SSCLC, treating cells during differentiation with 10 pM and 10 nM of araC significantly decreased *HTT* expression in HD SSCLC compared to WT SSCLC (Supplementary Information Fig. 7). When gene expressions of selected genes involved in DNA damage response (DDR) pathways were compared to no treatment group, WT iPSC showed induced expression of *ATR* and *FAN1* when it was treated with 200 mM caffeine while aspirin and araC treatment suppressed *DDB2* expression (Fig. 7a). WT iPSC showed induced expression of *DDB2* with caffeine, aspirin, and araC treatment, while aspirin suppressed *OGG1* expression and araC induced *PCNA* expression (Fig. 7b). HD1 iPSC showed suppressed DDR gene expression with 200 mM caffeine treatment (Fig. 7c). Treated with aspirin and araC, HD1 iPSC showed suppressed *OGG1* expression, and *ATR* expression was suppressed with aspirin treatment, while *ATM* and *MSH3* were suppressed with araC treatment (Fig. 7c). In HD1 SSCLC, DDR genes showed generally suppressed expression with aspirin and araC treatments (Fig. 7d). HD2 iPSC responded differently from WT iPSC and HD1 iPSC with caffeine, aspirin, and araC treatment (Fig. 7e). In HD2 SSCLC, except *OGG1*, most DDR genes did not change expression when treated with caffeine, and aspirin and araC suppressed *OGG1* and *FAN1* expression, and araC suppressed *ERCC5* while only 10 nM of araC suppressed *PCNA* and *RAD51* expression (Fig. 7f).

**Fig. 8:**
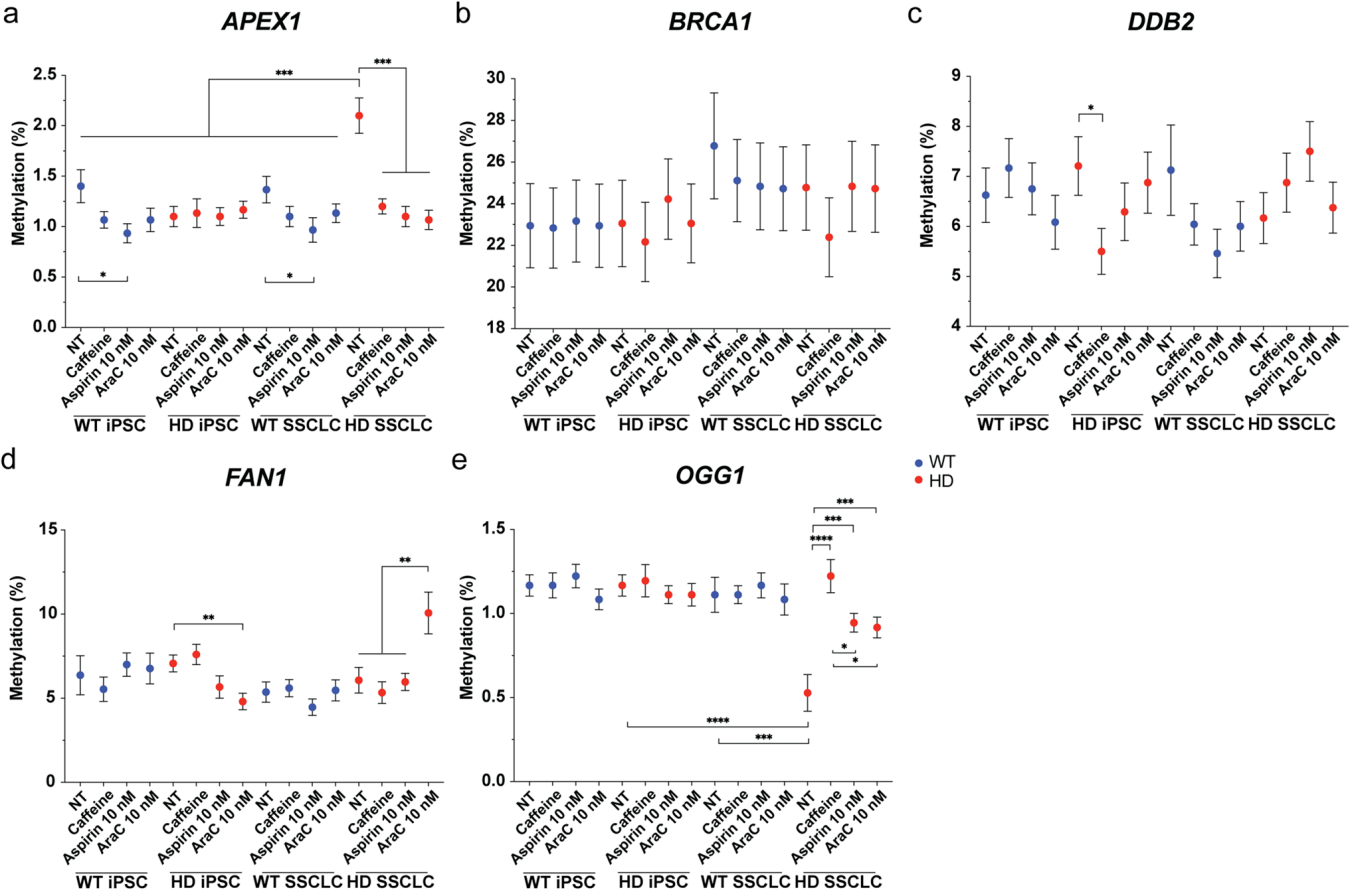
Differential methylation profile of selected genes involved in DDR.** A** In both iPSC and SSCLC, WT showed a significant decreased in methylation with 10 nM aspirin treatment (*p* = 0.0165 and *p* = 0.0295). Compared to all iPSC and WT SSCLC, HD SSCLC showed a significant increase in *APEX1* methylation. Compared to the HD iPSC no treatment group and WT SSCLC no treatment group, HD SSCLC showed a significant increase in methylation (*p* < 0.001 and *p* = 0.00142). Also, treatment with caffeine, aspirin, and araC all significantly decrease the methylation (*p* < 0.001, *p* < 0.001, and *p* < 0.001 respectively). **B** Methylation of *BRCA1* did not change after SSCLC differentiation and after the chemical treatment. **C** Methylation of *DDB2* was significantly decreased in HD iPSC treated with caffeine than the no-treatment group (*p* = 0.0264). **D** HD iPSC treated with araC showed a significant decrease in methylation compared to the no treatment group (*p* = 0.0020). Treating HD SSCLC with araC significantly increased methylation of *FAN1* compared to all other groups especially compared to no treatment group (*p* = 0.00802). **E** Compared to HD iPSC no treatment, HD SSCLC no treatment showed a significant decrease in methylation (*p* < 0.001). Also, HD SSCLC showed significantly lower methylation in *OGG1* compared to WT SSCLC (*p* < 0.001). Also, HD SSCLC no treatment showed lower methylation compared to caffeine, aspirin, and araC treatment group (*p* < 0.001, *p* = 0.00109, and *p* = 0.00272). HD SSCLC treated with caffeine showed significantly higher methylation than aspirin and araC treated groups (*p* = 0.0165 and *p* = 0.0104)

In human spermatogenesis, DNA methylation remodeling occurs continuously during the developmental process [29], and oxidative DNA damage, environmental exposure, and nutrition can change DNA methylation of sperm [18, 30–32]. To further investigate how the chemicals might have regulated those genes, methylation profiles of five genes that either showed a similar trend as TNR expansion suppression or have previously been reported to be associated with TNR expansion were analyzed by the pyrosequencing. *APEX1*, *BRCA1*, and *DDB2* were selected based on the gene expression that correlates with CAG repeat stabilization, and *OGG1* and *FAN1* have been associated with TNR expansion [33–36]. In both iPSC and SSCLC, WT showed significant decreased in methylation with 10 nM aspirin treatment (Fig. 8a). Compared to all iPSC and WT SSCLC, HD SSCLC showed a significant increase in *APEX1* methylation (Fig. 8a). Compared to HD iPSC no treatment group and WT SSCLC no treatment group, HD SSCLC showed a significant increase in methylation of *APEX1* (Fig. 8a). Also, treatment with caffeine, aspirin, and araC all significantly decrease the methylation (Fig. 8a). When HD1 and HD2 were analyzed separately, HD1 and HD2 showed a significant increase in methylation at *APEX1* (Supplementary Information Fig. 8). Both *BRACA1* and *DDB2* did not show a difference in methylation between cell type and chemical treatments (Fig. 8b, c). However, *DDB2* showed a decreased methylation in HD iPSC with 200 mM caffeine treatment compared to the no treatment group (Fig. 8c). *FAN1* showed no differential methylation in WT iPSC and WT SSCLC (Fig. 8d). HD iPSC treated with 10 nM of araC showed a significant decrease in methylation compared to the no treatment group (Fig. 8d). However, in HD SSCLC, SSCLC treated with 10 nM of araC showed a significant increase in methylation of *FAN1* compared to no treatment, 200 mM caffeine treated, and 10 nM aspirin-treated cells (Fig. 8d). Both HD1 and HD2 SSCLC showed a similar trend as they both showed increased *FAN1* methylation, but HD2 SSCLC showed a more significant change than HD1 (Supplementary Information Fig. 8). Both WT and HD showed no change in *OGG1* methylation in iPSC (Fig. 8e). However, HD SSCLC showed a significant decrease in methylation after the differentiation (Fig. 8e), and both HD1 and HD2 showed similar methylation changes after the differentiation (Supplementary Information Fig. 8). Also, HD SSCLC showed significantly lower methylation in *OGG1* than WT SSCLC (Fig. 8e). HD SSCLC with no treatment showed lower methylation compared to caffeine, aspirin, and araC treatment group (Fig. 8e). HD SSCLC treated with caffeine showed significantly higher methylation than aspirin and araC treated groups (Fig. 8e).

## Discussion

In Huntington’s disease, CAG repeat expansion occurs in male germlines [8–11]. Due to the limitation of availability and ethical concerns, a robust study to dissect the mechanisms involved in TNR instability in human spermatogenesis has been challenging. Understanding the mechanisms of CAG repeat expansion and developing drugs or therapies that can stabilize or even induce contraction of CAG repeat size in male germ cells will provide an opportunity to eradicate the devastating effect of genetic anticipation in trinucleotide repeat expansion disorders (TREDs). Mouse and other model organisms have been instrumental in discovering processes involved in TNR expansion. Still, differences in kinetic, biological, and developmental differences in the spermatogenesis process [37], limited availability, and ethical usage of human tissues leave room for developing a better system to study the paternal expansion of CAG repeat in spermatogenesis. To overcome the current limitations, we have developed an in vitro stem cell model to replicate TNR instability in spermatogenesis.

We have reported CAG repeat expansion in HD monkey lymphocytes and sperm [5] and monkey spermatogenic cells derived from embryonic stem cells in vitro [24]. The current study expands our previous study by utilizing HD patient-derived induced pluripotent stem cell (iPSC) to replicate TNR instability observed in sperm of HD patients that can provide a platform to study TNR instability and the underlying mechanism of genetic anticipation in human germ cells. As we have demonstrated in monkey ESC [24], we confirmed the expansion of CAG repeat in human iPSC-derived SSCLC in HD cells (Fig. 3, Supplementary Information Fig. 9). Although HD1 with the initial CAG repeat size of 14.72 Q and 38.88 Q showed a moderate but not statistically significant increase in CAG repeat size after the differentiation, HD2 with the initial CAG repeat size of 15.62 Q and 238 Q showed a statistically significant expansion after the differentiation (Fig. 4). These findings were consistent with previous studies in rodents [38–40], monkey [5, 24], and human [41–44], where larger CAG repeats are more unstable and prone for expansion.

In this study, we expanded our previous study on monkey SSCLC by treating differentiating SSCLC with two known TNR stabilizing agents, aspirin and araC [27]. While we showed HD2 iPSC was compared to SSCLC, both aspirin and araC prevented further expansion of CAG repeat size in HD2 SSCLC (Fig. 6d, Supplementary Information Fig. 4b). The moderate changes observed in HD1 might be also important because loss and gain of a single CAG repeat could delay or expedite onset by an average of 3 years in 40–50 Q range [45].

To investigate possible mechanisms involved in CAG repeat size change observed in this study, we evaluated the gene expressions of a selected panel of DNA damage response (DDR) genes by quantitative real-time PCR. As a preliminary study, changes in gene expressions in the selected panel of genes were analyzed to infer the involvement of a specific DDR pathway although further analysis is needed to relate those changes to steady-state levels of protein. In general, SSCLC showed induced expression of DDR genes in both WT and HD (Supplementary Information Fig. 5c, d). *OGG1*, 7,8-dihydro-8-oxoguanine-DNA glycosylase, initiates BER by recognizing and removing 8-oxoG from the opposite of C in DNA [46], which has been associated with the CAG expansion [34, 47]. The *OGG1* knockout (*OGG1*^−/−^) R6/1 mice showed suppression of age-dependent CAG repeat expansion [34]. Since increased CAG repeat size does not necessarily induce 8-oxo-G lesions, the authors of the study suggested that the 8-oxo-G lesions are favored within CAG sites, or *OGG1* binds to CAG repeat DNA tertiary structures [34]. Another gene involved in the BER pathway, *APEX1*, was also induced in HD SSCLC (Supplementary Information Fig. 5b). APEX1, apurinic/apyrimidinic endonuclease 1, has been reported to incise the 5′end of an abasic site in DNA and TNR hairpin loop [48, 49]. Also, *APEX1* can stimulate *OGG1, FEN1*, and *LIG1*, which can initiate BER [50–53]. Recent studies have associated oxidative stress with the expansion of TNR [34, 54–57].

Two genes, *FAN1* and *MSH3*, have recently been the focus of the HD research due to recent genome-wide association studies (GWAS) identifying those two genes as genetic modifiers of HD [58]. Increased expression of both *FAN1* and *MSH3* in SSCLC compared to iPSC will be interesting to investigate further in future studies. Although *FAN1* is involved in suppressing somatic repeat expansion, in germ cells, *FAN1* knockout has no effect on repeat expansion in fragile X disorders [59]. *MSH3* induces repeat instability in somatic cells in both HD and DM models [60–63], and in the germline, *Msh3* knockout suppressed expansion, and in DM, even promoted contraction [64, 65]. Also, *MSH3* expression levels are reported to be rate-limiting in the expansion mechanism [64, 65], but the contribution of *MSH3* expression level on repeat expansion in human spermatogenesis remains to be explored.

HD SSCLC specific induced expression of *NEIL1*, *ERCC5*, *XRCC1*, and *BRCA1* might be involved in further expansion of the already expanded CAG repeat. Of those genes, *Neil1* has been identified as a genetic modifier of the intergenerational repeat instability [66]. Recent studies have suggested crosstalk between BER and MMR [61, 67]. Although MMR plays a more critical role in somatic CAG repeat expansion in mouse models, the interaction between factors involved in BER or other DDR pathways is not well understood in human germline cells. These targets can be modified with siRNA or CRISPR-Cas9 in our in vitro culture model to investigate their function in CAG repeat expansion during human spermatogenesis.

*HTT* expression was induced in SSCLC, but *HTT* expression did not significantly change by neither aspirin nor araC treatments (Supplementary Information Fig. 7). Therefore, it is unlikely that TNR stabilization by aspirin or araC has resulted from the suppressed expression of *HTT*.

Aspirin was suspected that the induction of expression of MMR proteins, such as MLH1, MSH2, MSH6, and PMS2, by aspirin facilitates apoptosis [68]. The concentration of aspirin used in this study is well below the physiological concentration found in patients, which might not have been enough to induce dosage-response in this study (20 mM) [69]. Except for HD2 iPSC and WT SSCLC, aspirin suppressed most DDR gene expression (Fig. 7). In all SSCLC, the expression of *OGG1* and *FAN1* was significantly suppressed with aspirin treatment (Fig. 7b, d, e). Since *OGG1* is mainly involved in BER by removing 8-oxoG, the antioxidant activity of aspirin might explain the stabilization effect of aspirin on TNR. In human spermatozoa, the truncated BER pathway is functional, containing only OGG1 protein [70]. Reactive oxygen species (ROS) is necessary for basic sperm function [71, 72]. Guanine is the most susceptible to oxidation, and ROS endogenously generates 8-oxoG. The CAG sites could be more prone to 8-oxoG lesions, and CAG repeats tertiary structures might bind better with OGG1 [34]. The oxidation-excision-expansion cycle that is present and accumulates in the brain might also be presented in spermatogenesis.

Except in WT SSCLC, araC suppressed most of DDR gene expression as well although the mechanism of action is different from aspirin (Fig. 7). Cytosine arabinoside (araC, cytarabine, 1-b-D-arabinofuranosylcytosine) is a cytosine analog nucleoside that competes with deoxycytidine triphosphate (dCTP) and blocks the DNA replication process [73]. Also, araC inhibits DNA polymerase δ, the primary polymerase involved in the DNA mismatch repair [74], while allowing translesion DNA synthesis (TLS) polymerase η [75]. AraC inhibits the DNA synthesis stage of MMR [27], and it is possible that araC [76] treatment have changed the preference between replication DNA polymerases and translesion DNA polymerase and mediate TNR repair when hairpin or loop structure is formed during DNA replication. Like aspirin, the suppression of *FAN1* in SSCLC might suggest that oxidative stress response is more involved in CAG repeat expansion in spermatogenic cells rather than MMR identified as genetic modifiers involved in CAG repeat expansion in fibroblast and blood samples.

HD SSCLC showed a significant increase in *APEX1* methylation after the differentiation, and HD2 showed a more significant increase than HD1 (Fig. 8a, Supplementary Information Fig. 8). In contrast, *OGG1* showed a significant decrease in methylation in HD after the differentiation (Fig. 8d). In BER, APEX1 displaces OGG1 from apurinic/apyrimidinic (AP) sites generated by the OGG1 glycosylase activity allowing the base excision repair to proceed [77], which suggest induced activity of OGG1 in SSCLC cause induced BER activity, and restoring methylation back to the WT range of both *APEX1* and *OGG1* by aspirin and araC treatments could have helped to suppressed TNR expansion in SSCLC. The methylation of *FAN1* was only significantly induced in HD SSCLC when treated with araC (Fig. 8d), and only HD2 SSCLC showed induced *FAN1* methylation with araC treatment (Supplementary Information Fig. 8), which correlates to gene expression profile (Fig. 7f). Although the involvement of FAN1 in somatic repeat expansion is well-documented, FAN1 did not show any effect on repeat expansion in germ cells in a fragile X mouse model [59]. However, in fragile X, germline expansion occurs mainly through the female lineage [3], and *Fan1* has been reported to be involved in non-canonical mechanisms to control somatic CAG instability in the mice [78]. Therefore, genomic context, cell-type, and human-specific functions of *FAN1* involvement in human germline expansion need further investigations.

Our data suggest that in vitro derived SSCLC can replicate dynamic CAG repeat insatiability and genetic anticipation. Studies have identified genetic modifiers that might be involved in germline TNR instability, including *NEIL1* [66], *MSH2* [79], *MSH3* [64, 65], *MSH6* [64], *FEN1* [80], *DNMT1* [81]*,* and *CBP* [82]. However, none of the studies have utilized the human system to assess the impact of those genetic modifiers in germline TNR instability. The majority of polyglutamine protein products function in DNA repair, and the growing number of expanded repeat diseases suggest shared genetic modifiers [83–86]. Many TREDS shares similar characteristics such as pathogenic repeat threshold, negative correlation between age of onset and repeat size, somatic and germline expansion, and similar genetic modifiers [87]. These facts suggest that underlying mechanisms of CAG expansion might share the same mechanism, and our platform can be used to investigate germline TNR instability in other TREDs.

As a monogenic dominant disorder, HD can be accurately diagnosed through preimplantation genetic testing (PGT). The recent development of sequencing technologies allows accurate diagnosis of monogenic disorders (PGT-M), chromosomal structural rearrangements (PGT-SR), and aneuploidy (PGT-A) [88]. Also, the improved efficiency and safety of PGT guarantee the prevention of transmission of HD. If the patient does not wish to undergo pre-symptomatic testing, preimplantation exclusion genetic testing can be conducted to prevent HD transmission. Exclusion testing is indirect, and it is based on haplotype genetic markers from the grandparents. However, even the embryo that is inherited haplotypes from the affected grandparent only has a 50% risk of carrying CAG expansion which can lead to serious moral and ethical objections [89]. Moreover, because CAG expansion occurs during spermatogenesis, people who do not have family members with HD can still have children with HD. With the rapid technological advances in genetic testing, it is now possible for people to find out their HTT genotypes, and people with reduced penetrance range (36–39 CAG repeats) and intermediate repeats (27–25 CAG repeats) can take a precautionary approach my suppressing TNR expansion.

## Methods

### hiPSC culture

To investigate if in vitro spermatogenesis can replicate CAG instability in hiPSCs, two WT hiPSC lines (BJ and ND41658) and two HD iPSCs (ND38547 and ND36999) were used for in vitro spermatogenesis using established protocol [16]. Two HD cell lines carry different CAG repeat sizes: ND38547 carries 44 Q (HD1), and ND36999 carries 180 Q (HD2). BJ was gift from C.A.E. and all other cell lines were purchased through NINDS Stem Cell Repository. hiPSC lines were maintained in mTeSR™ Plus (STEMCELL Technologies, Canada) following the instruction provided by the manufacturer.

### Directed differentiation of hiPSC to spermatogenic cells

hiPSCs were differentiated to advanced spermatogenic cells by following our established protocol [90–93]. Briefly hiPSCs were cultured for 10 days in human spermatogonia stem cells (SSC) medium containing the following: minimum essential medium (MEM) alpha (Invitrogen), 0.2% bovine serum albumin, 5 mg/ml insulin, 10 mg/ml transferrin, 60 mM putrescine, 2mML-glutamine (Invitrogen), 50 mM b-mercaptoethanol, 1 ng/ml human basic FGF (hbFGF; BD Biosciences), 20 ng/ml GDNF (R&D Systems), 30 nM sodium selenite, 2.36 mM palmitic acid, 0.21 mM palmitoleic acid, 0.88 mM stearic acid, 1.02 mM oleic acid, 2.71 mM linoleic acid, 0.43 mM linolenic acid, 10 mM HEPES, and 0.5X penicillin/streptomycin (Invitrogen). The medium was changed every other day.

### Chemical exposures during the spermatogenesis process

During the 10-day differentiation process, cells were challenged with different concentrations of chemicals to determine the impact on CAG instability during the spermatogenesis process (Supplementary Information Fig. 10). The concentrations were based on the previous publications demonstrating physiological concentrations (urinary and blood): 10 pM and 10 nM for araC (Sigma) and aspirin (Sigma) [90–93] and 200 mM of caffeine [27]. The chemicals were added when the differentiation of iPSC media was changed.

### Immunocytochemistry

Cells were fixed using 4% paraformaldehyde (PFA) for 45 min and permeabilized using 0.2% Triton-X solution for 10 min. Cells were blocked for 1 h at room temperature using 1% normal horse serum and 5% BSA in PBS. After the initial blocking, primary antibodies were added and incubated overnight at 4 °C. Primary antibodies were washed away by washing cells with PBS three times, and secondary antibodies were added for 1 h at room temperature. The vendor and dilution information can be found in Supplementary Table 3. Images were captured by Olympus BX63 and using CellSens imaging software.

### RNA-seq data analysis

A total of 100,000 cells were harvested and washed with PBS twice to remove any debris. Cell pellets were snap-frozen and submitted to Yerkes National Primate Research Center Genomics Core for further analysis. Briefly, total RNA was isolated using Qiagen RNeasy Mini kit, and the quality control was conducted on 4200 Bioanalyzer Capillary electrophoresis (Agilent). The total RNA (10 ng) was used for mRNA amplification using Clontech Smarter V4 chemistry following the manufacturer’s protocol. Amplified mRNA was fragmented and barcoded using Illumina’s Nextera XT kits. Amplified Libraries were validated by Agilent 3200 Tapestation, and quantification was conducted on a Qubit Fluorimeter. The libraries were normalized, pooled, and clustered on Illumina HiSeq3000/4004 Flowcells using the Illumina cBOT. The prepared libraries were sequenced on an Illumina HiSeq3000 system in 101-base single-read reactions. The raw (FASTQ) files were uploaded to the Galaxy web platform, and data analysis was conducted on the public server at usegalaxy.org [94]. The sequence was mapped using Spliced Transcripts Alignment to a Reference (STAR) [95] (v2.6.0b-1) utilizing the latest release of human Ensembl genome assembly (hg38). Read counts were measured using featureCount [96] (v2.0.1). Differential expression analyses of two cell types were performed using DESeq2 method [97] (Galaxy Version 2.11.40.6 + galaxy1). The resulting *p*-values were adjusted with Benjamini and Hochberg method (FDR) with the threshold set at 0.05. Gene set enrichment (GSE) was conducted using the Ensemble of Genes Set Enrichment Analysis (EGSEA) (Galaxy Version 1.10.0) [98]. Differential expression analysis data and normalized read counts were used to generate graphs in RStudio (1.4.1717) running on R Package (R-4.1.0).

### qRT-PCR

Briefly, for quantification of mRNA expression, mRNA was extracted using Trizol® (Invitrogen) followed by DNA digestion using Turbo DNA-free™ Kit. RNA sample (500 ng) was reversed transcribed using iScript™ cDNA Synthesis Kit (Bio-Rad) and quantified using SYBR Green Supermix (Bio-Rad) using primers described in Supplementary Table 2 on CFX96 Real-Time Detection System (Bio-Rad). PCR conditions for SYBR Green assays are as followed: Initial 95 °C for 30 s, followed by 39 cycles of 95 °C for 10 s and 55 °C for 20 s with melt curve analysis.

### DNA extraction

DNA from cell pellets were extracted using the DNeasy Extraction Kit (Qiagen), and the concentration and purity of each sample were determined using NanoDrop™ 2000 (Thermo Fisher, Waltham, MA).

### Assessment of CAG instability

CAG instability was be analyzed by GeneScan analysis. Day 0 was used to establish the baseline for TNR instability in spermatogenesis for each cell line. TNR instability was assessed by PCR as previously described [8]. Briefly, DNA was extracted from cell pellets by adding lysis buffer (0.5% Tween-20, 0.1 mg proteinase K, 1X PCR buffer (Takara)). The reaction was incubated at 56 °C for 45 min followed by 95 °C for 10 min. PCR reaction was carried out using 50 ng of DNA. Two primers, forward Hu4F, 5′-FAM-atggcgaccctggaaaagctgatgaa, and reverse HD5R, 5′-cggctgaggcagcagcggctgt, which flanks the CAG repeat tract, were used to amplify the CAG repeat region. PCR conditions as followed: 98 °C for 5 min, followed by 40 cycles of 96 °C for 5 min, 67 °C for 45 s, and 72 °C for 1.5 min, and 72 °C for 10 min. Each reaction contained MgCl_2_ (2 nM), 1% of Betain, and 0.05 U Taq polymerase (Takara). PCR products were run on 2% agarose gel to verify the product presence, and 10 uL of the product was submitted for GeneScan analysis at Emory Integrated Genomics Core. The samples were run on ABI 3130xl Genetic Analyzer (ABI). The precise size of the product was calculated against GeneScan™ 1000 ROX™ dye Size Standard (ABI). The sizing of PCR fragments was conducted using GeneMarker Software Version 2.7.0 (Softgenetics®).

### Data analysis using curve fitting method

The data were analyzed using GeneMarker® (SoftGenetics); allele data was imported into MatLab (MathWorks). We employed a previously described method [99], which has been used in multiple publications [100, 101]. Briefly, imported masked data were analyzed using ipf.m function in MatLab optimizing for error (less than 10%) and overall fitness R^2^ value (higher than 0.95). Prolonged in vitro culture has been reported to induce TNR contraction [102–104] and expansion [105–109]. Also, in order to minimize the PCR amplification bias toward smaller alleles, and possible DNA synthesis errors known as “PCR stutter,” all peaks that cross the threshold were analyzed in this study. The consecutive normal Gaussian distribution was used to fit the data maximizing for the coverage area of the raw data set. Later, electrograms and curve-fit results were combined using Adobe Photoshop and Adobe Illustrator (Adobe). Expansion index was calculated by modifying instability index [110] following the equation: $\Sigma \left(\left(\frac{\mathrm{peak\ height}}{\mathrm{\Sigma peak\ heights}}\right)\left(\Delta TNR\ \mathrm{ from\ the\ reference\ allele}\right)\right)$. This represents the instability of a sample and its tendency toward expansions (i.e., positive values) or contractions (i.e., negative values). Expansion index close to 0 indicates low instability, while values between 1 and 5 show medium instability and values over 5 show high instability. For each cell type (iPSC and SSCLC), no treatment group was used to define the reference alleles. The numerical changes in TNR sizes are presented in Supplemental Table 1. Expansion index lacks the ability to separate the continuous and periodic expansion of trinucleotide repeats. Therefore, both the curve-fit method and expansion index methods were used in this study. For the linear regression, the Pearson correlation coefficient (*R*^2^) and statistical significance (*p*-value) were calculated using GraphPad Prism (GraphPad). For statistical significance, a *p*-value of less than 0.05 and an *R*^2^ value of larger than 0.95 were used.

### Pyrosequencing

The methylation profiles of *APEX1*, *BRCA1*, *DDB2*, *FAN1*, and *OGG1* were assessed with pyrosequencing. Isolated DNA was bisulfite-treated with EpiTect Fast DNA Bisulfite Kit (Qiagen). Bisulfite-treated DNA samples were assessed on the PyroMark Q48 System using PyroMark CpG primers (Qiagen) described in Supplementary Table 4.

### Ethics approval

This study was approved by the Institutional Review Board (IRB) and the Biosafety Committee of Emory University.

## Conclusion

Investigating CAG repeat instability in spermatogenesis is challenging due to biological differences among different animal models and the limited availability of human HD testicular tissues [1, 13, 38, 111–114]. We have developed an in vitro model to replicate CAG repeat instability utilizing patient-derived human pluripotent stem cells to address such challenges and provide unique insights. This proof-of-concept study demonstrates an in vitro system to study TNR instability in human spermatogenesis. In this study, we demonstrate CAG repeat expansion in the in vitro spermatogenesis system and suppressed expansion of TNR during spermatogenesis by aspirin and araC treatment. However, we only have analyzed the gene expressions of a panel of selective genes and DNA methylation changes; thus, a further study is needed to address how these changes at the genomic level can impact at the protein level. Our study presents an alternative to study CAG repeat instability and provides a platform to evaluate novel therapeutics that can be used to stabilize or even induce contraction in HD spermatogenic cells.

## Supplementary Information

ESM 1 (DOCX 66 kb)

ESM 2 (PNG 4631 kb)

High resolution image (TIF 34322 kb)

ESM 3 (PNG 727 kb)

High resolution image (TIF 47053 kb)

ESM 4 (PNG 1271 kb)

High resolution image (TIF 22425 kb)

ESM 5 (PNG 157 kb)

High resolution image (TIF 5514 kb)

ESM 6 (PNG 107 kb)

High resolution image (TIF 7916 kb)

ESM 7 (PNG 369 kb)

High resolution image (TIF 12878 kb)

ESM 8 (PNG 76 kb)

High resolution image (TIF 16576 kb)

ESM 9 (PNG 152 kb)

High resolution image (TIF 9633 kb)

ESM 10 (PNG 155 kb)

High resolution image (TIF 8943 kb)

ESM 11 (PNG 79 kb)

High resolution image (TIF 3429 kb)

## Declarations

### Disclaimer

This work was prepared while Anthony W.S. Chan was employed at Yerkes National Primate Research Center and Emory University. The opinions expressed in this article are the author’s own and do not reflect the view of the National Institutes of Health, the Department of Health and Human Services, or the US government.
