# Supplementary material for: Suppression of trinucleotide repeat expansion in spermatogenic cells in Huntington’s disease
Source: J Assist Reprod Genet. 2022 Sep 6;39(10):2413–30. doi: 10.1007/s10815-022-02594-x (PMC9596677; doi:10.1007/s10815-022-02594-x)
Supplement: Supplementary file 1 — (DOCX 66 kb) [file 10815_2022_2594_MOESM1_ESM.docx]

**Supplemental Data**

**Supplemental Table 1**. Change in TNR of HD1 and HD2 before and after SSCLC differentiation compared to no treatment group.

|  | HD1 | | | | HD2 | | | | | | |
| --- | --- | --- | --- | --- | --- | --- | --- | --- | --- | --- | --- |
| Treatment | iPSC | | SSCLC | | iPSC | | | | SSCLC | | |
|  | 14Q | 40Q | 14Q | 40Q | 18Q | 35-200Q | >200Q | 18Q | | 35-200Q | >200Q |
| Caffeine | -0.7558 | 0.1441 | -0.07925 | -0.7103 | 1.832 | 10.62 | 15.87 | 0.7934 | | -28.86 | 11.54 |
| Aspirin 10 pM | -1.333 | -1.975 | -0.2886 | -1.559 | 1.483 | -0.4042 | 0.1833 | -0.4719 | | -48.64 | -16.55 |
| Aspirin 10 nM | -1.452 | -1.997 | -0.7667 | -2.4 | -1.338 | -11.54 | -3.117 | 0.531 | | -47.99 | -13.94 |
| AraC 10 pM | -0.03467 | -2.204 | -0.7311 | -2.275 | 0.6337 | 5.819 | -8.471 | 0.4865 | | -36.46 | -6.031 |
| AraC 10 nM | -1.029 | -1.167 | -0.7521 | -0.4202 | 1.301 | -17.62 | -7.646 | 0.4243 | | -24.45 | -38.28 |

**Supplemental Table 2**. List of primers used in this study for RT-PCR.

| **Gene Name** | **Primer Name** | **Sequence** |
| --- | --- | --- |
| *OCT4* | hOCT4-F | GACAGGGGGAGGGGAGGAGCTAGG |
|  | hOCT4-R | CTTCCCTCCAACCAGTTGCCCCAA AC |
| *NANOG* | hNANOG-F | AGTCCCAAAGGCAAACAACCCACT TC |
|  | hNANOG-R | TGCTGGAGGCTGAGGTATTTCTGTC TC |
| *VASA* | hVASA (DDX4)-F | TGCTGGTGAGTGTAATAAGCGA |
|  | hVASA (DDX4)-R | TTCCATCTCGATAGCCGCC |
| *DAZL* | hDAZL-F | TTAAACTTCGAGGGCCGGAG |
|  | hDAZL-R | TTTGCAGTAGACTCGTGCGG |
| *ZBTB16* | hZBTB16-F | CGGTTCCTGGATAGTTTGC |
|  | hZBTB16-R | GGGTGGTCGCCTGTATGT |
| *GFRA1* | hGFRA1-F | CCAAAGGGAACAACTGCCTG |
|  | hGFRA1-R | CGGTTGCAGACATCGTTGGA |
| *ACR* | hACR-F | GTGGCTGTTGTACGTGAAGA |
|  | hACR-R | GAAGTCGCAGACGAAGGA |
| *TNP1* | hTNP1-F | ACAAGTGGGAGCGGTAA |
|  | hTNP1-R | TAGTCCACCACCAAAGCG |
| *GAPDH* | hGAPDH-F | AATCCCATCACCATCTTCC |
|  | hGAPDH-R | CATCACGCCACAGTTTCC |
| *HTT* | hHTT Exon1 F | GTGCTGAGCGGCGCCGCGAGTC |
|  | hHTT Exon1 R | GGACTTGAGGGACTCGAAGGC |
| *APEX1* | APEX1-qPCR-F | CAATACTGGTCAGCTCCTTCGG |
|  | APEX1-qPCR-R | TGTTACCAGCACAAACGAGTCA |
| *BRCA1* | BRCA1-qPCR-F | GGTTGTTGATGTGGAGGAGCAA |
|  | BRCA1-qPCR-R | AGGCTGATTCCAGATTCCAGGT |
| *MSH3* | MSH3-qPCR-F | GAATGTCTGGCAACTCTGAGCC |
|  | MSH3-qPCR-R | GACTCTACTTTGAGGAAGGGCAG |
| *XRCC1* | XRCC1-qPCR-F | CTGAAGAGACCAAAGCAGCCTC |
|  | XRCC1-qPCR-R | CCCATTGTCCTGTCCTTCTGAC |
| *OGG1* | OGG1-qPCR-F | GACCAACAAGGAACTGGGAAACT |
|  | OGG1-qPCR-R | GGCAGAAGATAAGAGGACGCAG |
| *FEN1* | FEN1-qPCR-F | CTGAGAAGGGAGAGCGAGCTTA |
|  | FEN1-qPCR-R | AACACAGAGGAGGGATGACTGG |
| *NEIL1* | NEIL1-qPCR-F | GCAGTTCAGGGAGAATGTGCTA |
|  | NEIL1-qPCR-R | TGCCATTGAAGAACCTCTGGTC |
| *DDB2* | DDB2-qPCR-F | CATCAAAGGGATTGGAGCTGGA |
|  | DDB2-qPCR-R | CTTGCAGCCTAGTTGTTCCCTC |
| *ATR* | ATR-qPCR-F | GGGCTCCCTTCTCAGTCATGTA |
|  | ATR-qPCR-R | AATCTTGCACAGCATCCCTGTT |
| *ATM* | ATM-qPCR-F | GAGTGCAGTGACAGTGATGTGT |
|  | ATM-qPCR-R | CATGTTCTAGTTGACGGCAGCA |
| *ERCC5* | ERCC5-qPCR-F | ACTTAAAGGAGTCCGGGATCG |
|  | ERCC5-qPCR-R | CTGGAGTCACTGGACGCTAA |
| *LIG1* | LIG1F | AAAGTGCTGGACAGGAAGGG |
|  | LIG1R | CAGGCTTCTGGCCTTTAGCA |
| *RAD51* | RAD51F | AGTGGCTGAGAGGTATGGTCT |
|  | RAD51R | AGAAGCATCCGCAGAAACCT |
| *PCNA* | PCNAF | AAGAGGAGGAAGCTGTTACCAT |
|  | PCNAR | TCCTCGATCTTGGGAGCCA |
| *FAN1* | FAN1F | CCTTGTGGTGCTGAAAACCG |
|  | FAN1R | TGCAACTCAGATTCTGTCTGT |

**Supplemental Table 3**. List of antibodies and dilution used in this study.

| **Antibody** | **Host** | **Vender** | **Cat #** | **Clone** | **Dilution** |
| --- | --- | --- | --- | --- | --- |
| VASA | Mouse | Santa Cruz | SC-517247 | 2F9H5 | 1:250 |
| ACR | Rabbit | Santa Cruz | SC-67151 | H-40 | 1:800 |
| DAZL | Mouse | Santa Cruz | SC-390929 | E6 | 1:100 |
| PIWIL2 | Rabbit | Santa Cruz | SC-377347 | D-5 | 1:500 |
| ZBTB16 | Mouse | R&D | MAB2944 | 6318100 | 1:1000 |
| OCT4 | Mouse | Santa Cruz | SC-5279 | C-10 | 1:500 |
| Alexa 488 | D@Mouse IgG (H+L) | ThermoFisher | A32766 |  | 1:1000 |
| Alexa 488 | D@Rabbit IgG (H+L) | ThermoFisher | A32790 |  | 1:1000 |
| Alexa 594 | D@Mouse IgG (H+L) | ThermoFisher | A32744 |  | 1:1000 |
| Alexa 594 | D@Rabbit IgG (H+L) | ThermoFisher | A21203 |  | 1:1000 |

**Supplemental Table 4**. PyroMark primer information used in this study

| **Pyro** | **Cat No.** | **Lot No.** | **T_a_** |
| --- | --- | --- | --- |
| Hs_APEX1_PyroMark CpG | PMC00089927 | 362309238 | 53°C |
| Hs_OGG1_03_PM | PM00013776 | 362309236 | 53°C |
| Hs_DDB2_01_PM | PM00046907 | 362309237 | 53°C |
| Hs_BRCA1_02_PM | PM00064869 | 362309235 | 53°C |
| Hs_MTMR15_01_PM | PM00060487 | 364812881 | 53°C |

**Supplementary Figure 1**. Differentiation confirmation of WT1 (A,B), WT2 (C,D), HD1 (E,F) and HD2 (G,H) with immunofluorescence. Representative immunofluorescence showing expression of spermatocyte marker (PIWIL2) (A’, C’, E’, and G’), spermatogonial stem cell marker (PLZF/ZBTB16) (A”, C”, E”, and G”), spermatid marker (ACR) (B’, D’, F’, and H’), and primordial germ cell marker. (DAZL) (B”, D”, F”, and H”) (scale = 100 μm, insert scale = 50 μm).

**Supplementary Figure 2.** Representative electropherogram of WT cells. **A** Electropherograms of WT1 iPSC with different chemical treatments. **B** Electropherograms of WT2 iPSC with different chemical treatments. **C** Electropherograms of WT1 SSCLC with different chemical treatments. **D** Electropherograms of WT2 SSCLC with different chemical treatments.

**Supplementary Figure 3.** Representative electropherogram of HD cells. **A** Electropherograms of HD1 iPSC with different chemical treatments. **B** Electropherograms of HD2 iPSC with different chemical treatments. **C** Electropherograms of HD1 SSCLC with different chemical treatments. **D** Electropherograms of HD2 SSCLC with different chemical treatments. *Inlets show a magnified portion of the electropherogram.

**Supplementary Figure 4. Expansion index of all treatment groups. A** Chemical treatment of HD1 before and after the differentiation did not show statistically significant changes. **B** Small allele from all SSCLC treatment group showed significantly increased expansion index compared to iPSC. AraC 10 nM treatment of iPSC showed significantly lower expansion index compared to caffeine and aspirin (10 pM and 10 nM) treatment group (p = 0.01059, p = 0.0347, and p = 0.0488 respectively). Compared to all treatment groups before differentiation, SSCLC no treatment control showed increased expansion index after the differentiation for the larger allele (200-300 Q, purple circles). With chemical treatments, both aspirin and araC treatment showed significant decrease in expansion index compare to no treatment control in SSCLC for all concentration groups (p = 0.00753 for aspirin 10 pM, p = 0.0377 for aspirin 10 nM, p = 0.00118 for araC 10 pM, and p = 0.000007 for araC 10 nM). Also, higher concentration of araC (10 nM) showed significant decrease in expansion index compared to lower 10 pM concentration of araC (p = 0.0001). The larger intermediate alleles (75-199 Q) showed significant decrease in expansion index when SSCLC no treatment group was compared to both no treatment and caffeine treatment group (p = 0.0107 and p = 0.00944). Both aspirin concentration of 10 pM and 10 nM showed reduced expansion index compared to no treatment group (p = 0.00286 and p = 0.00296 respectively). AraC treatment with 10 nM increased expansion index compared to no treatment (p = 0.0281) and both concentration of aspirin of 10 pM and 10 nM (p = 0.00249 and p = 0.00264). For all groups, mean and the standard error were plotted (n = 3). Multiple unpaired t-tests were conducted to calculate for the statistical significance. *p < 0.05, **p < 0.005, ***p < 0.0005, ****p < 0.00005.

**Supplementary Figure 5. Selected panel of DNA damage response (DDR) genes expression analysis by quantitative real-time PCR. A** When WT and HD iPSC were compared, most of DDR genes showed similar gene expression except *ERCC5*, *DDB2*, and *BRCA1*. *ERCC5* and *DDB2* showed significantly up-regulation (p = 0.00219 and p = 0.00207) while *BRCA1* showed down-regulations (p = 0.0238). **B** Few of DDR genes showed up-regulation in HD SSCLC compared WT SSCLC. Genes involved in inducing DNA damage response, *ATM* and *ATR*, were both up regulated in HD SSCLC (p = 0.042 and p = 0.0143). Genes involved in base excision repair (BER), *APEX1*, *OGG1*, and *XRCC1* were up regulated (p = 0.00216, p = 0.000037, and p = 0.0295). Also, *ERCC5*, *DDB2*, and *PCNA* showed up-regulation in HD SSCLC (p < 0.000001, p = 0.00361, and p < 0.000001). **C** When WT iPSC and WT SSCLC were compared, *OGG1*, *FAN1*, *MSH3*, *RAD51*, *LIG1*, and *APEX1* were up regulated (p = 0.000541, p = 0.0207, p = 0.0110, p = 0.0499, p = 0.0159, and p = 0.0313) while *DDB2* and *PCNA* were down regulated (p = 0.00544 and p = 0.0241). **D** Most of DDR genes were up regulated in HD SSCLC compared to HD iPSC except *DDB2*, *FEN1*, and *PCNA*. Genes up regulated in HD SSCLC were *ATM*, *ATR*, *NEIL1*, *OGG1*, *ERCC5*, *FAN1*, *MSH3*, *XRCC1*, *BRCA1*, *RAD51*, *LIG1*, and *APEX1* (p = 0.000019, p = 0.00850, p = 0.0151, p < 0.000001, p = 0.00385, p = 0.0123, p = 0.00148, p = 0.00147, p = 0.000082, p = 0.000002, p = 0.00401, p = 0.0395, and p = 0.000111). Multiple unpaired t-tests were conducted to calculate for the statistical significance (n = 6 to 12 based on the sample availability). *p < 0.05, **p < 0.005, ***p < 0.0005, ****p < 0.00005.

**Supplementary Figure 6. Quantitative real-time PCR data of DNA damage response genes in iPSC and SSCLC. A** When all WT and HD cells were analyzed together, SSCLC showed induced expression of DDR gene expression compared to iPSC except *ERCC5, DDB2, FEN1,* and *PCNA*. *DDB2* and *PCNA2* showed significantly reduced expression. **B** When WT and HD were compared separately, HD showed more significantly induced DDR gene expressions compared to WT except for *DDB2* and *PCNA*, which showed a significant reduction in both WT and HD. **C** When HD1 (44Q) and HD2 (180Q) were separately analyzed, HD1 generally showed higher induced DDR gene expression while HD2 showed significantly reduced expression of *FAN1* compared to control. **D** In SSCLC, both HD1 and HD2 showed similar induced expression of DDR genes. However, HD2 showed more similar DDR gene expression as WT except for *OGG1*, *ERCC5*, *DDB2*, and *PCNA*. (n = 3 to 6 based on the sample availability). *p < 0.05, **p < 0.005, ***p < 0.0005, ****p < 0.00005.

**Supplementary Figure 7. Huntingtin gene, *HTT*, expression comparison.** In general, the gene expression of HTT was significantly higher in SSCLC compared to iPSC for all differentiation and chemical treatment groups. Treatment of 200 mM of caffeine significantly induced *HTT* expression HD iPSC compared to WT iPSC (p = 0.00067). In SSCLC, treating cells during differentiation with 10 pM and 10 nM of araC significantly decreased *HTT* expression in HD SSCLC compared to WT SSCLC (p = 0.000152 and p = 0.0248). The mean and the standard error were plotted (n = 3 to 12 based on the sample availability). Multiple unpaired t-tests were conducted to calculate the statistical significance. *p < 0.05, **p < 0.005, ***p < 0.0005, ****p < 0.00005.

**Supplementary Figure 8.** Differential methylation profile of selected genes involved in DDR of HD1 and HD2. Both HD1 and HD2 showed similar methylation changes after the differentiation. HD1 and HD2 showed a significant increase in methylation at *APEX1* after the differentiation*.* Both HD1 and HD2 SSCLC showed a similar trend as they both showed increased *FAN1* methylation, but HD2 SSCLC showed a more significant change than HD1. Also, HD2 SSCLC showed induced *FAN1* methylation with araC treatment.

**Supplementary Figure 9.** Spermatogenic cell-type-specific gene expression profile of hiPSC (three right columns) and SSCLCs (six left columns) by 3-gene expression signature set (Hermann et al. 2018).

**Supplementary Figure 10.** Experimental design.
