# Supplementary figures and images for: Suppression of trinucleotide repeat expansion in spermatogenic cells in Huntington’s disease
Source: J Assist Reprod Genet. 2022 Sep 6;39(10):2413–30. doi: 10.1007/s10815-022-02594-x (PMC9596677; doi:10.1007/s10815-022-02594-x)

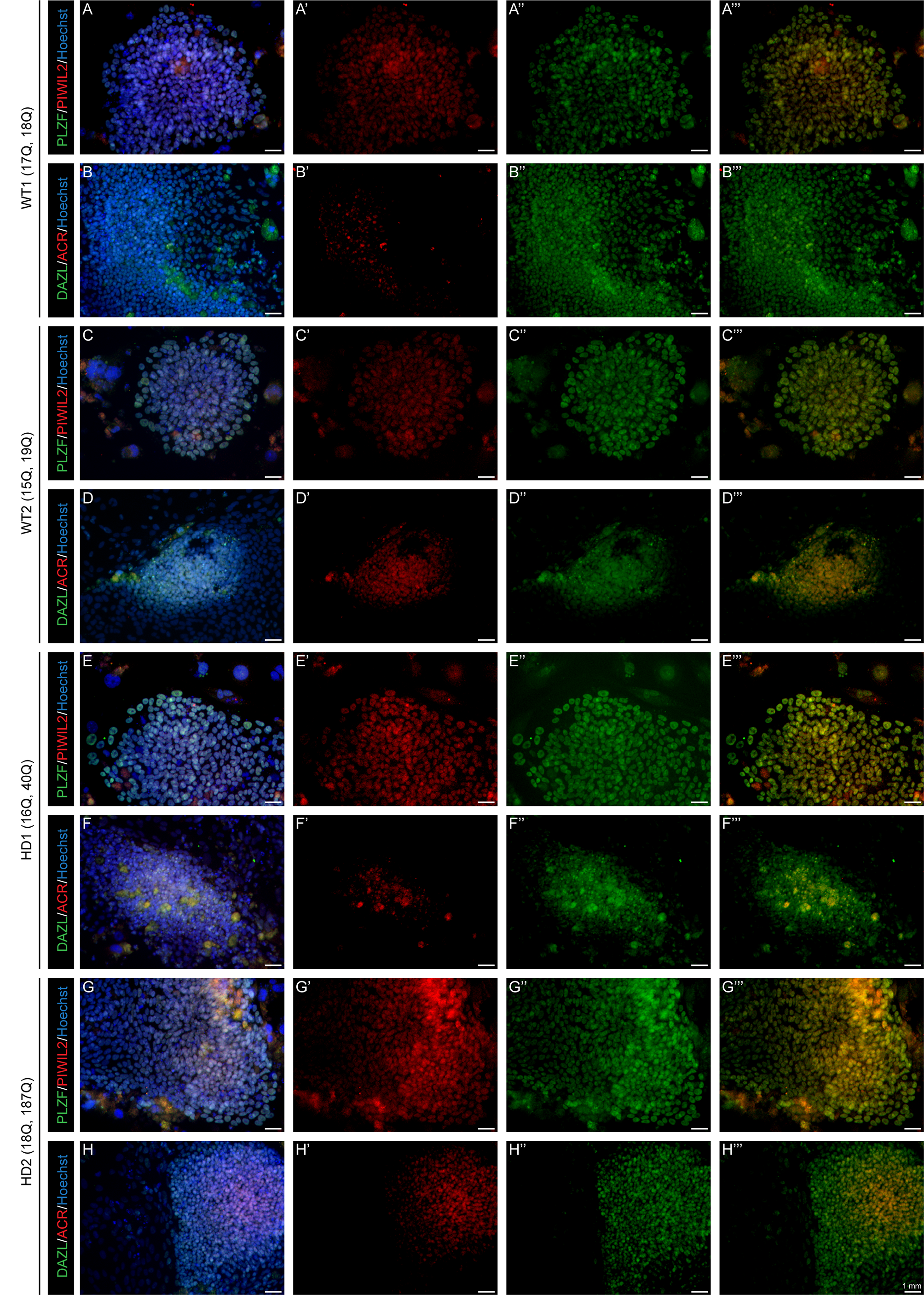

Supplement: Supplementary file 2 — (PNG 4631 kb) [file 10815_2022_2594_Fig9_ESM.png]

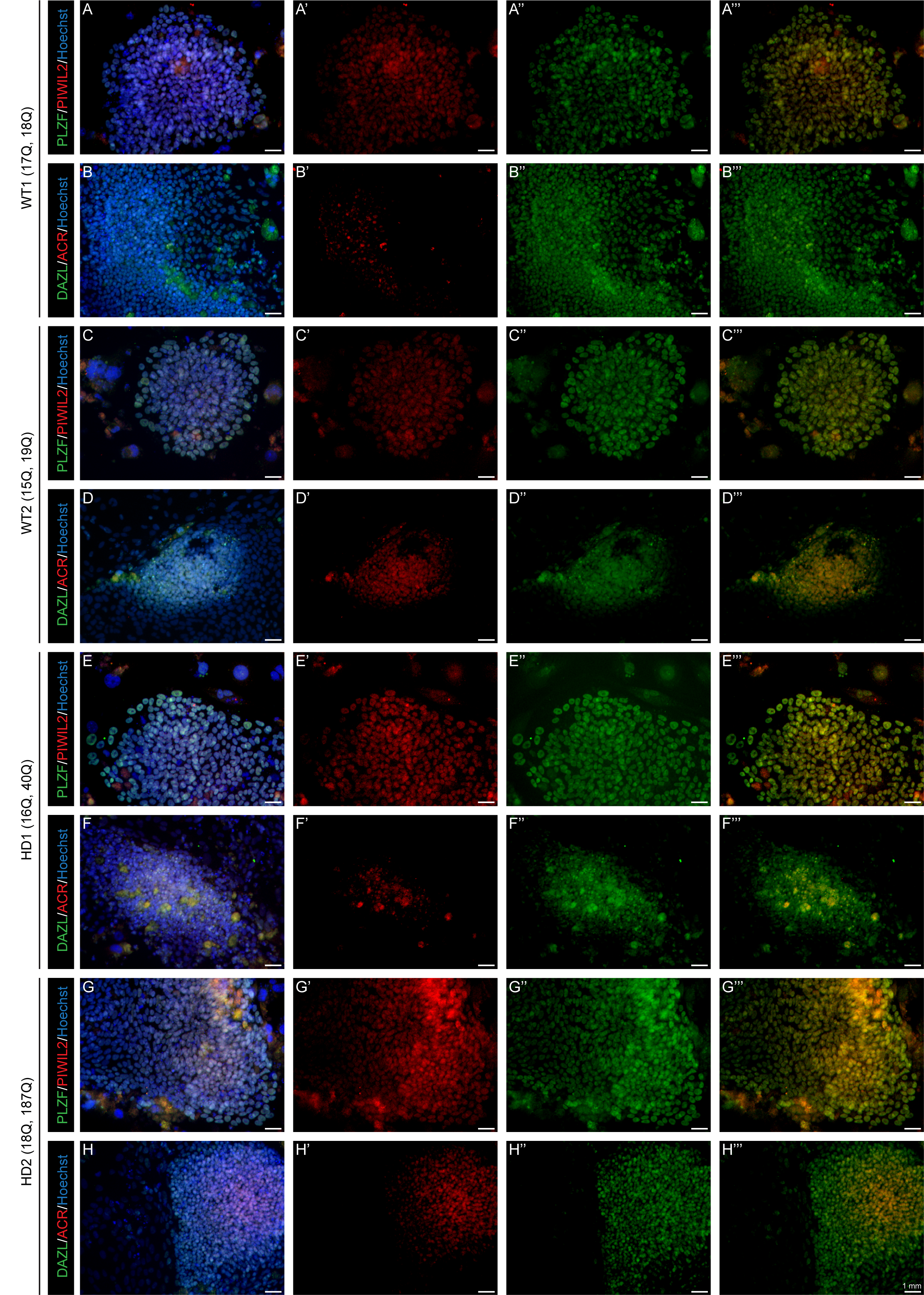

Supplement: Supplementary file 3 — High resolution image (TIF 34322 kb) [file 10815_2022_2594_MOESM2_ESM.tif]

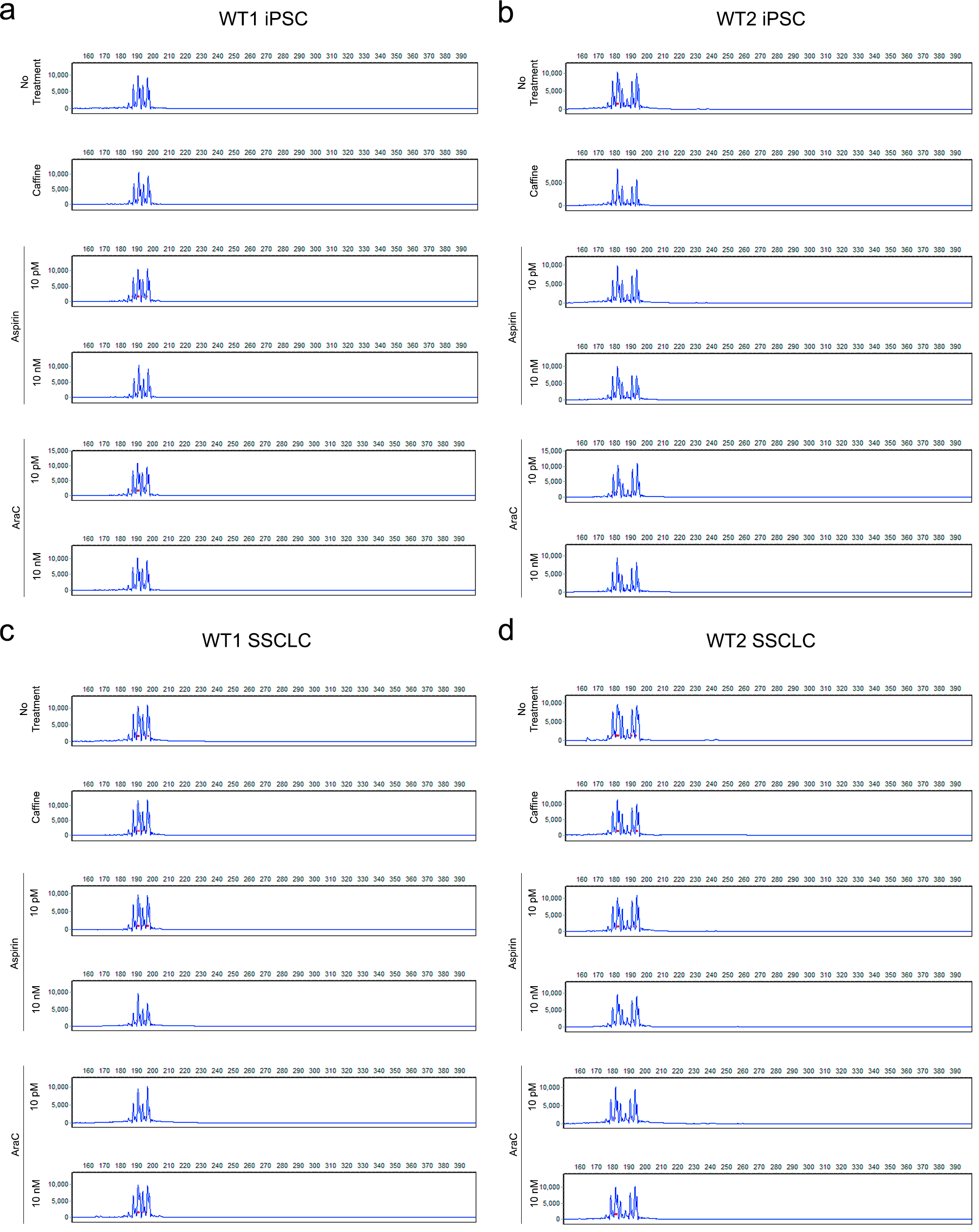

Supplement: Supplementary file 4 — (PNG 727 kb) [file 10815_2022_2594_Fig10_ESM.png]

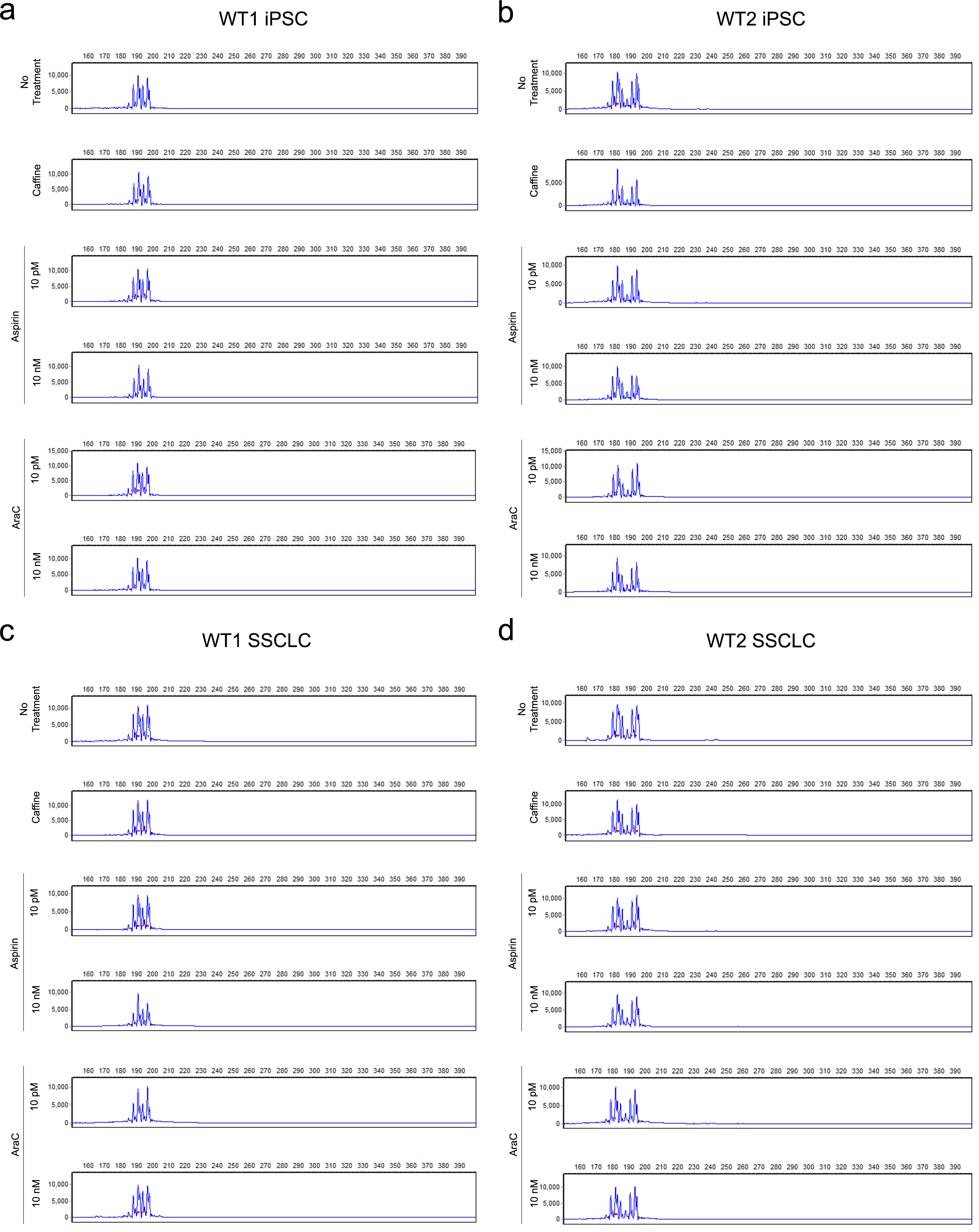

Supplement: Supplementary file 5 — High resolution image (TIF 47053 kb) [file 10815_2022_2594_MOESM3_ESM.tif]

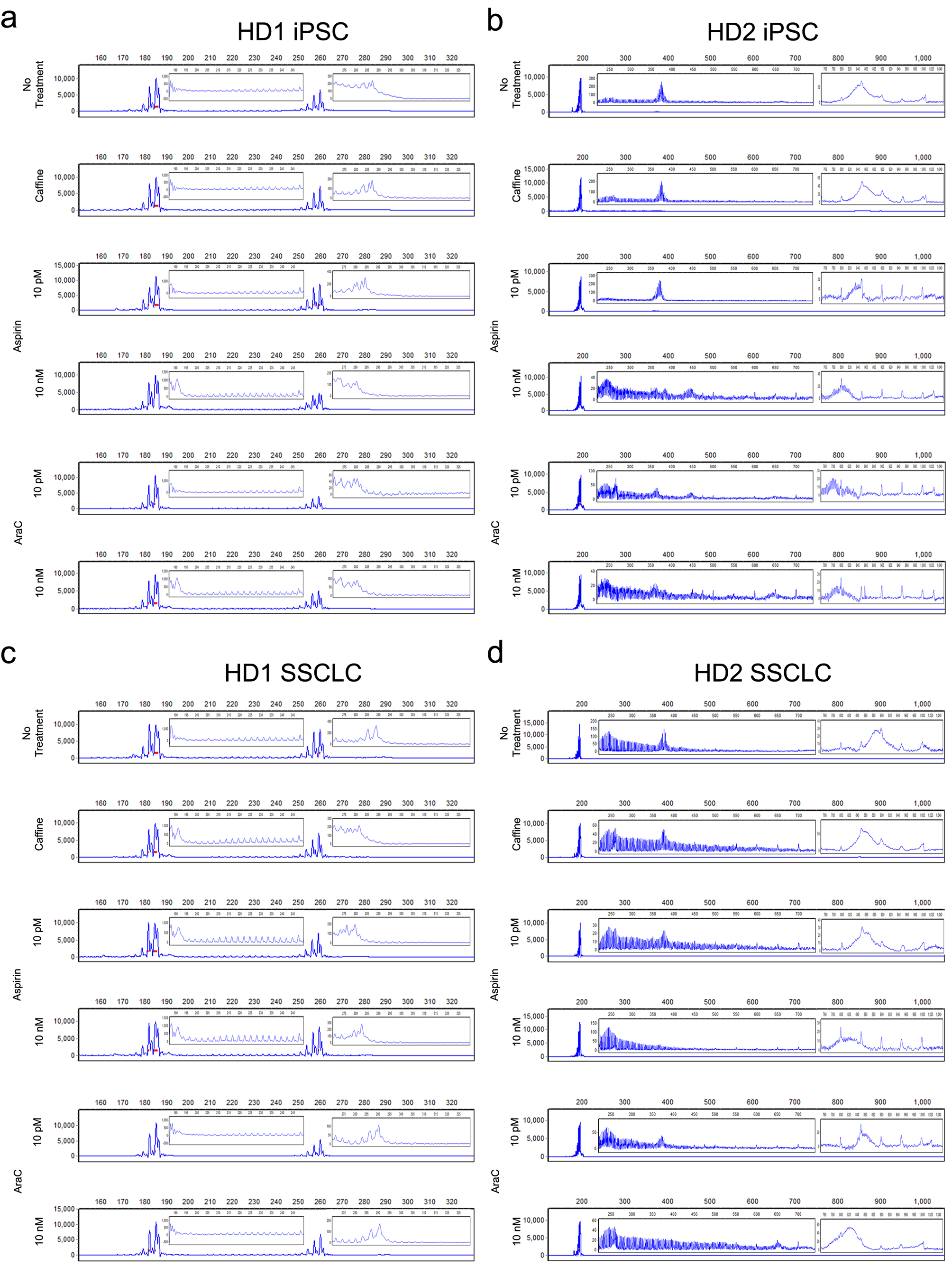

Supplement: Supplementary file 6 — (PNG 1271 kb) [file 10815_2022_2594_Fig11_ESM.png]

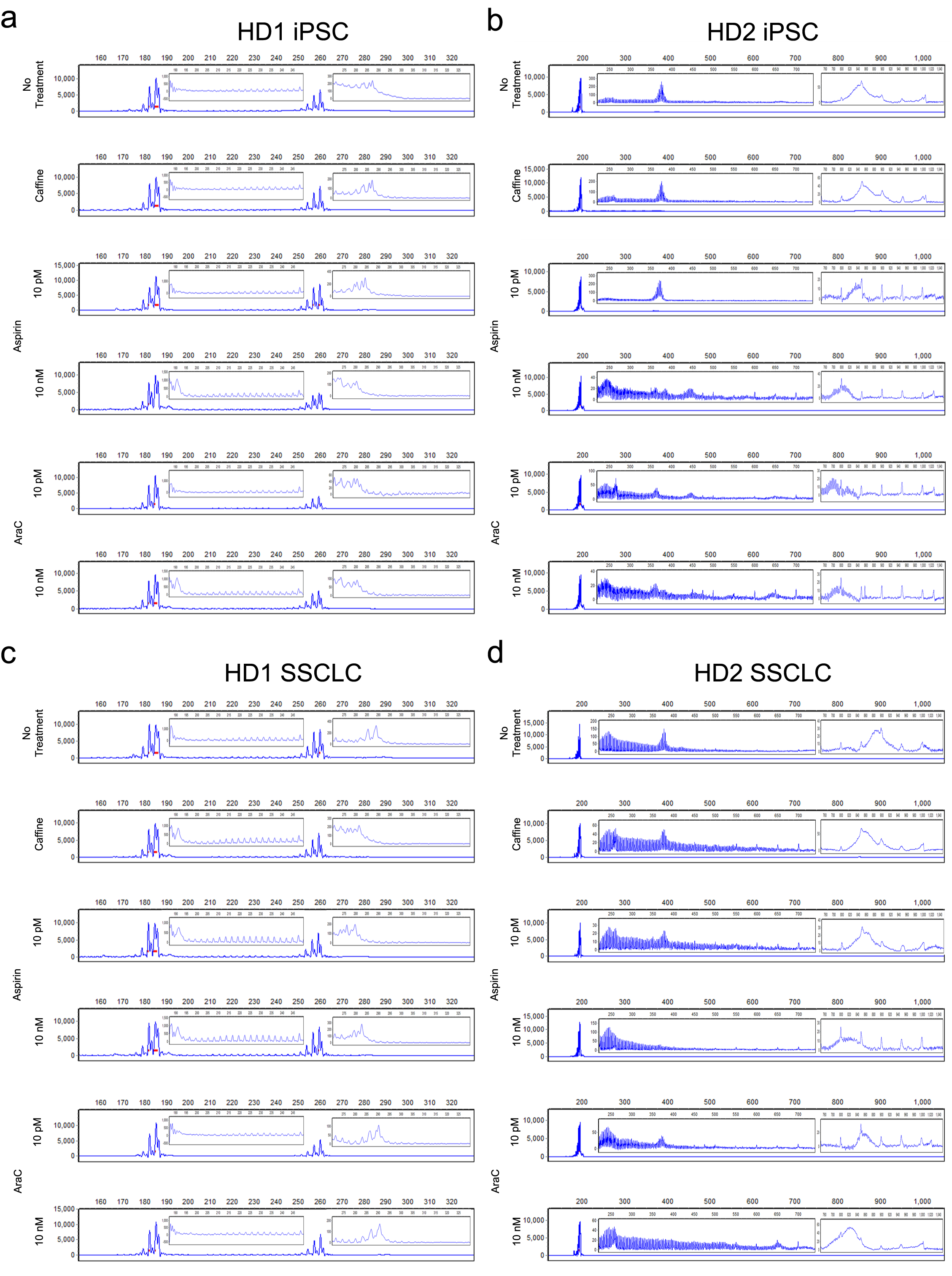

Supplement: Supplementary file 7 — High resolution image (TIF 22425 kb) [file 10815_2022_2594_MOESM4_ESM.tif]

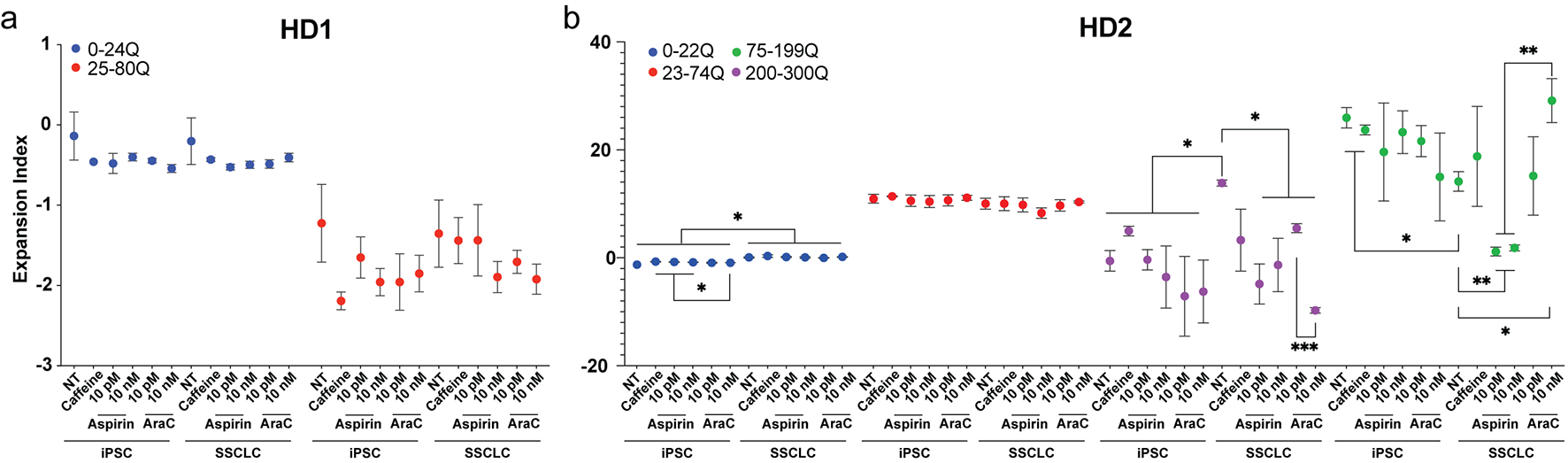

Supplement: Supplementary file 8 — (PNG 157 kb) [file 10815_2022_2594_Fig12_ESM.png]

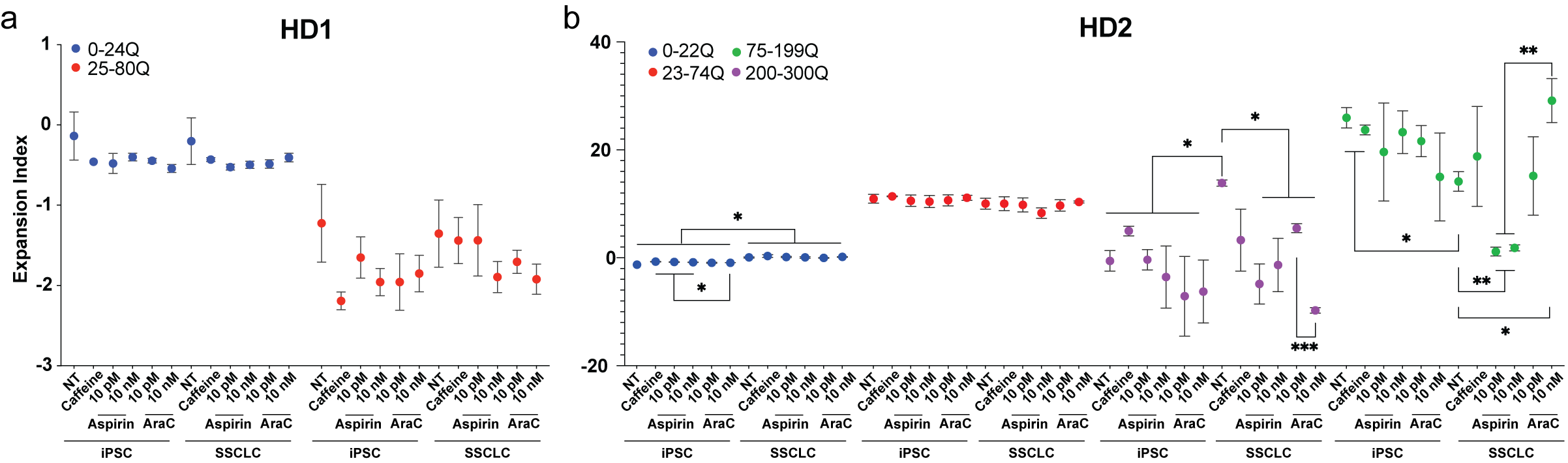

Supplement: Supplementary file 9 — High resolution image (TIF 5514 kb) [file 10815_2022_2594_MOESM5_ESM.tif]

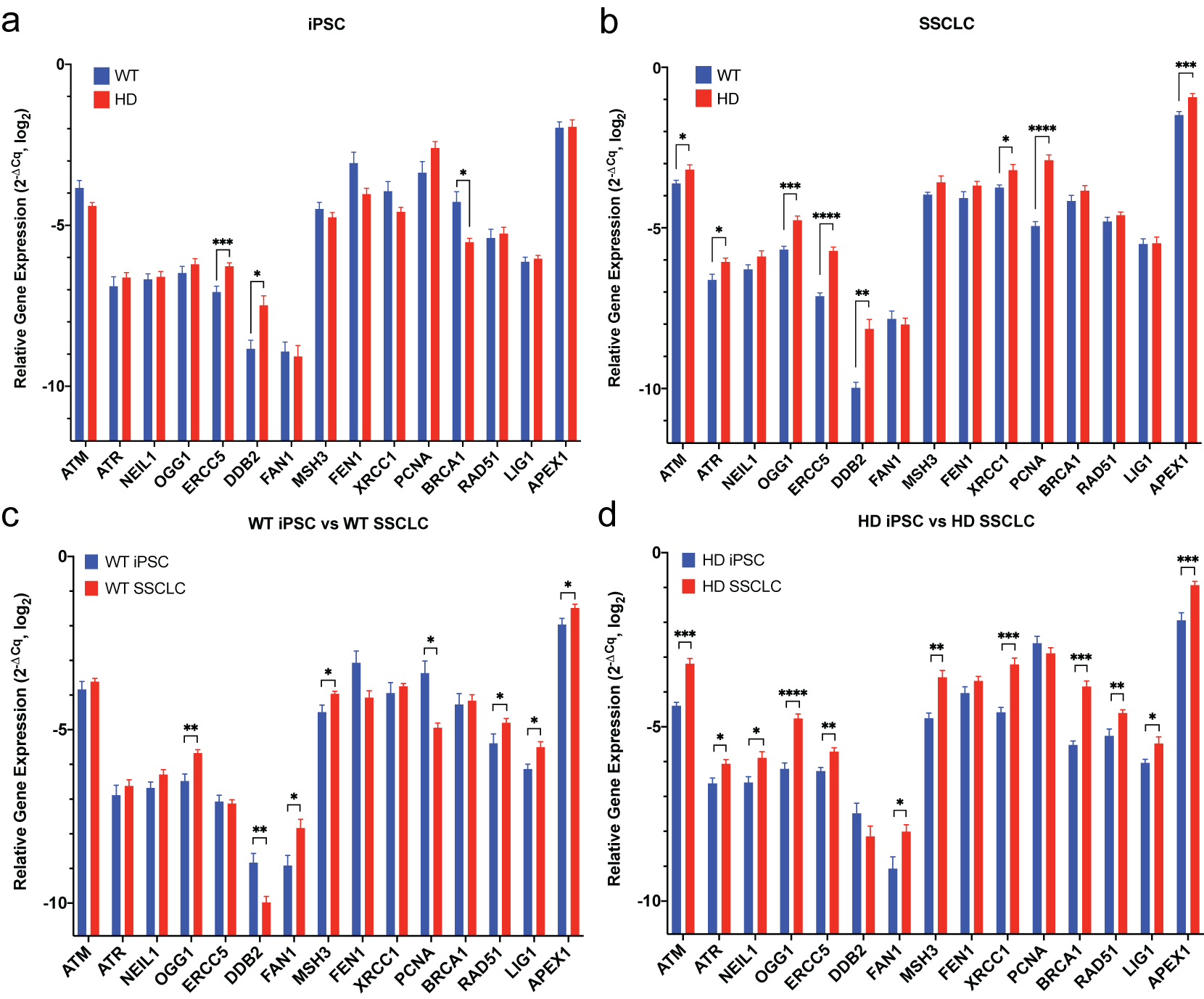

Supplement: Supplementary file 10 — (PNG 107 kb) [file 10815_2022_2594_Fig13_ESM.png]

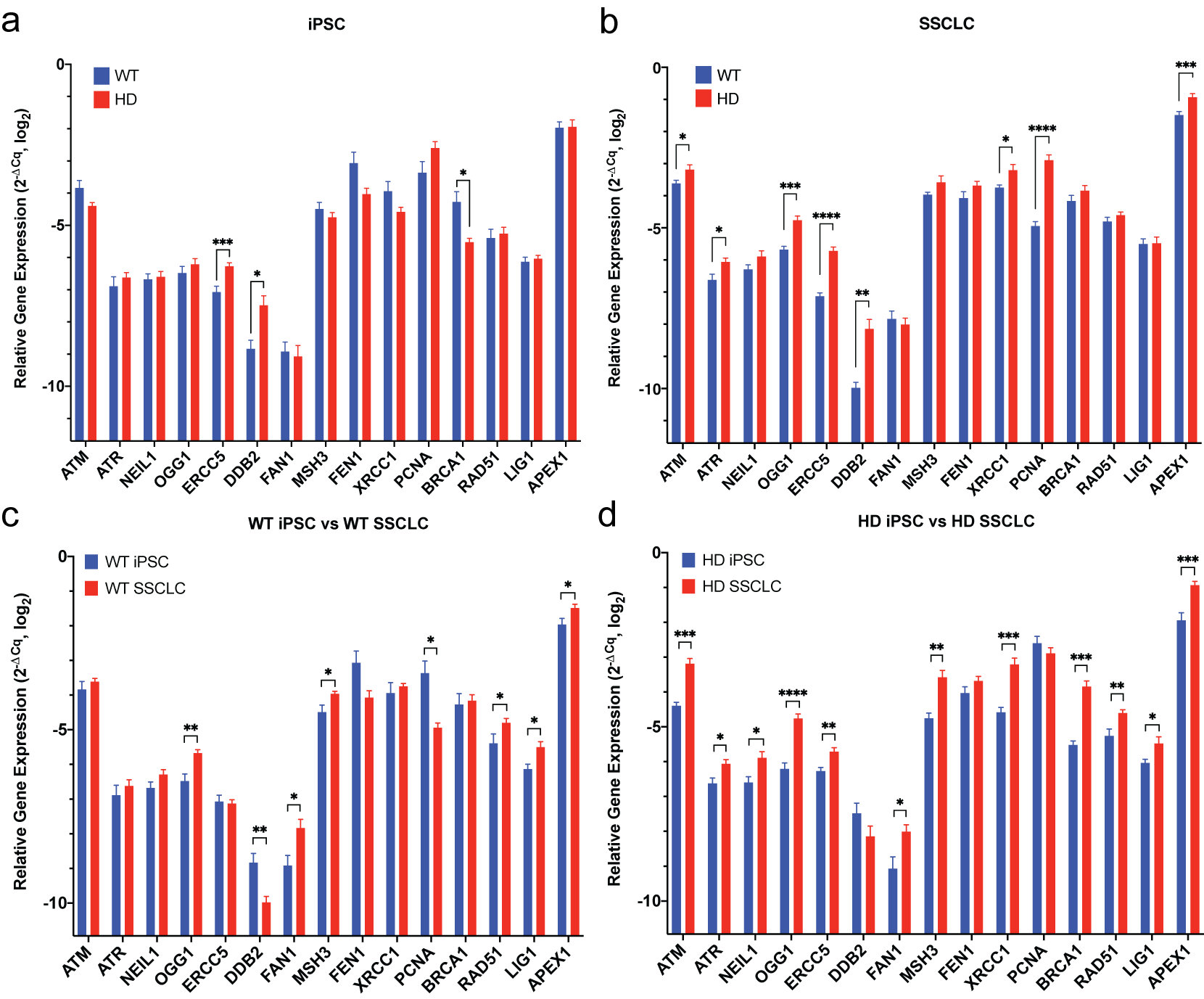

Supplement: Supplementary file 11 — High resolution image (TIF 7916 kb) [file 10815_2022_2594_MOESM6_ESM.tif]

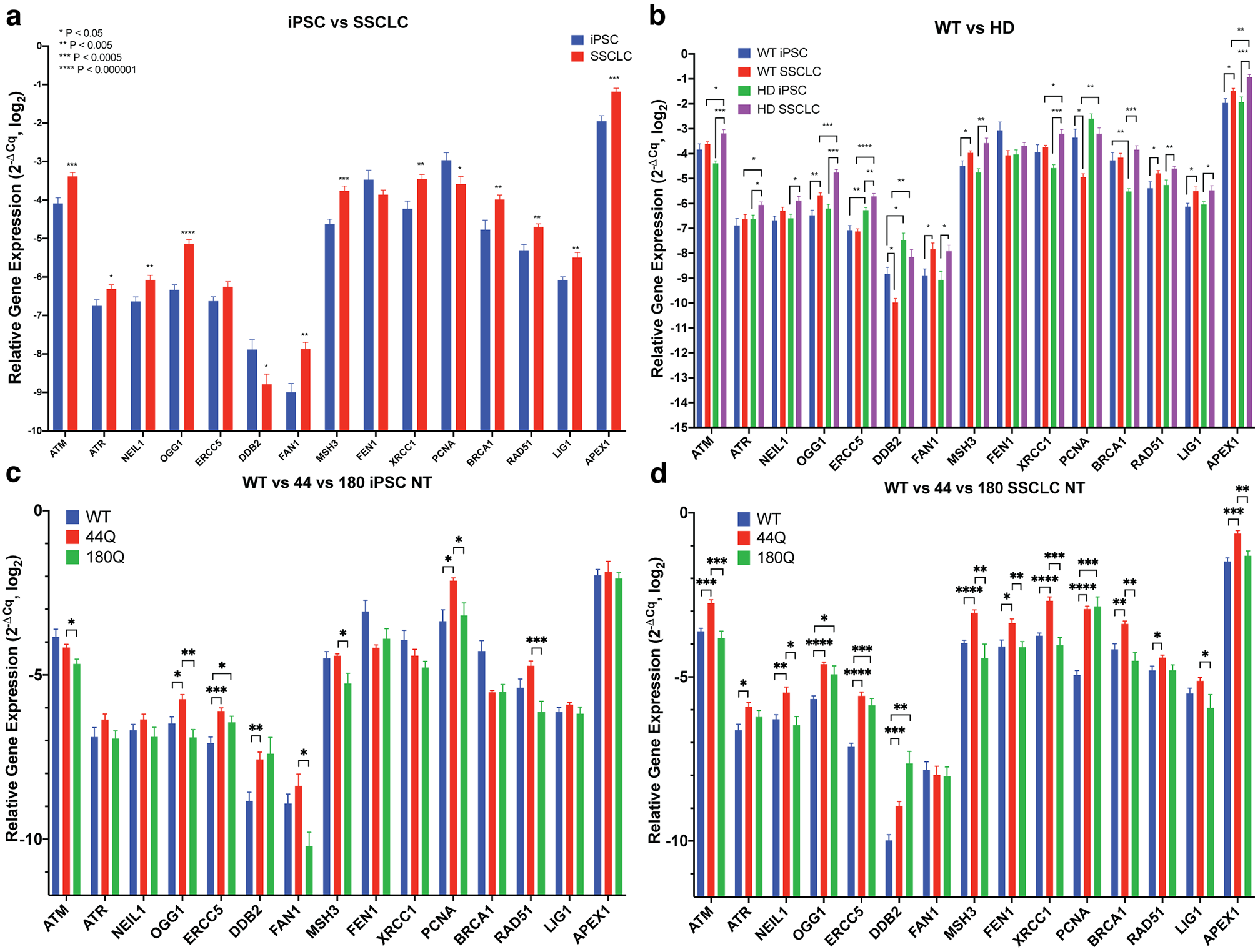

Supplement: Supplementary file 12 — (PNG 369 kb) [file 10815_2022_2594_Fig14_ESM.png]

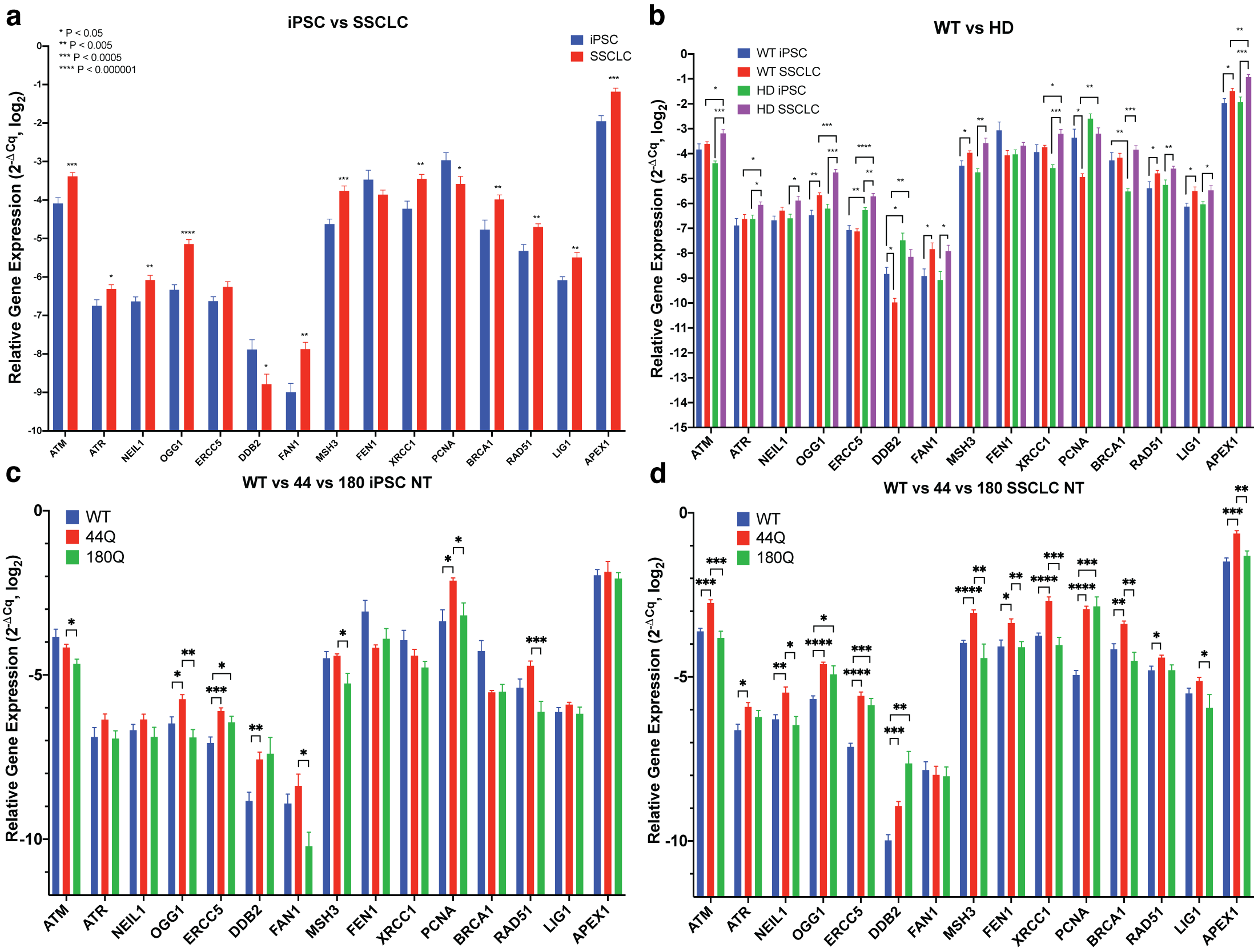

Supplement: Supplementary file 13 — High resolution image (TIF 12878 kb) [file 10815_2022_2594_MOESM7_ESM.tif]

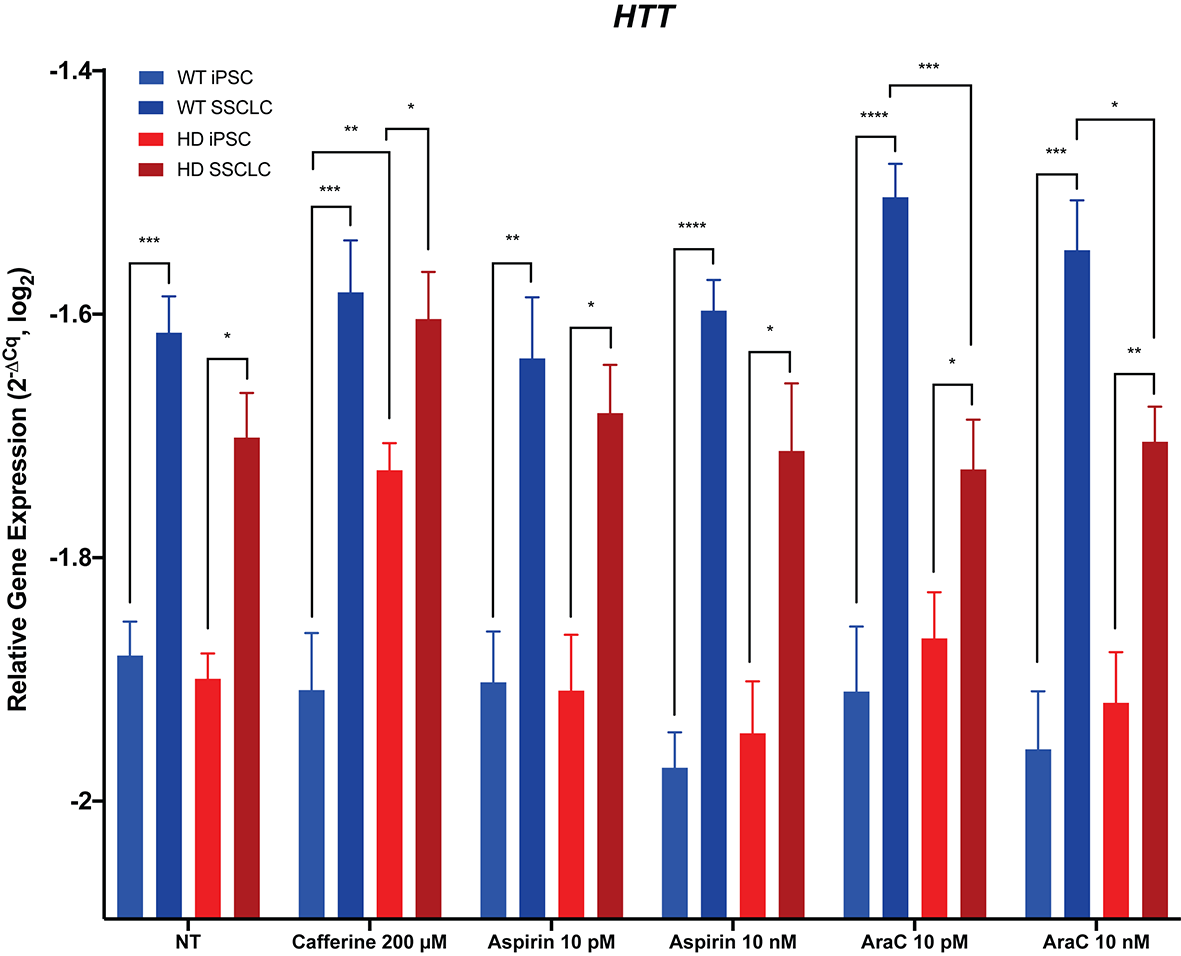

Supplement: Supplementary file 14 — (PNG 76 kb) [file 10815_2022_2594_Fig15_ESM.png]

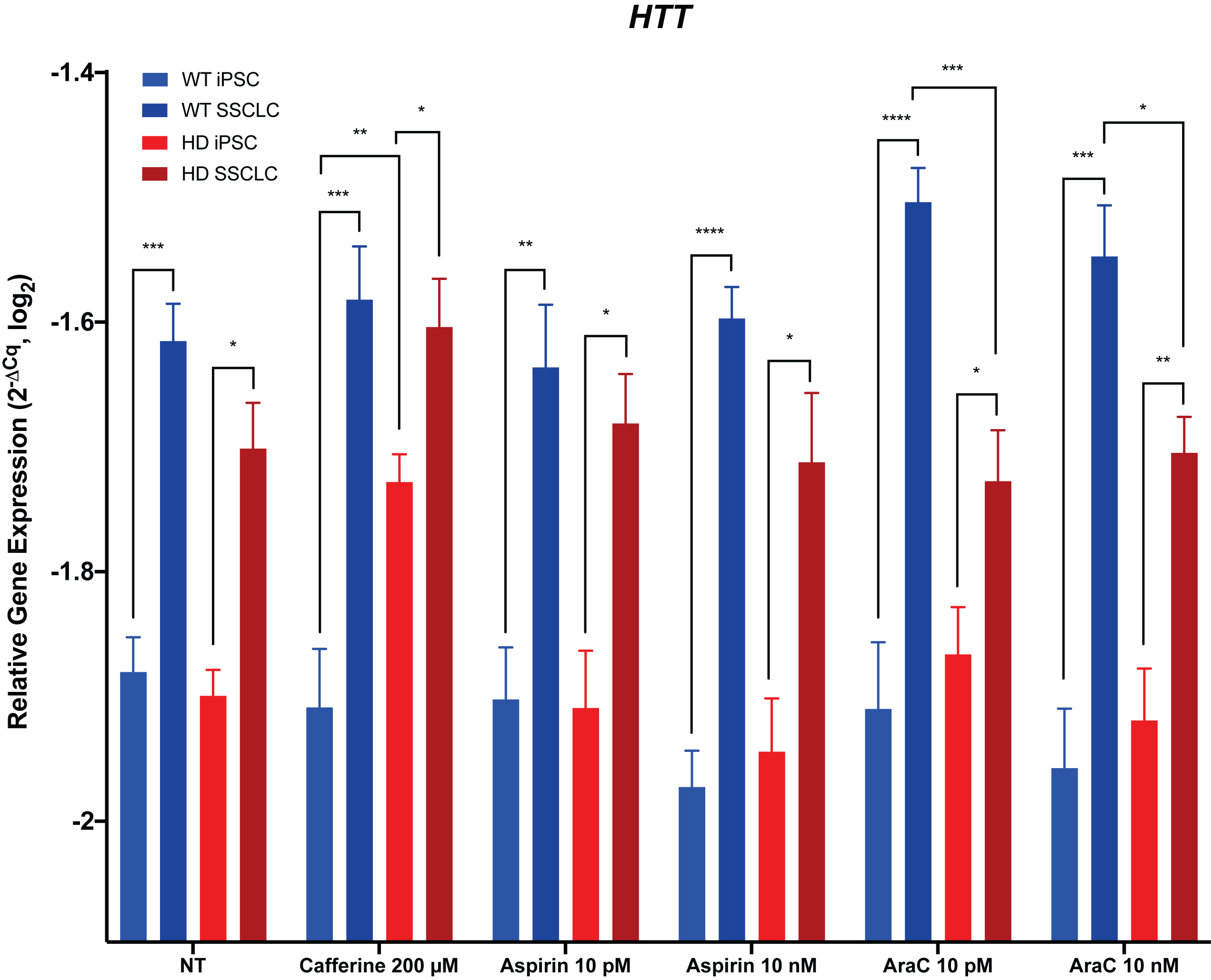

Supplement: Supplementary file 15 — High resolution image (TIF 16576 kb) [file 10815_2022_2594_MOESM8_ESM.tif]

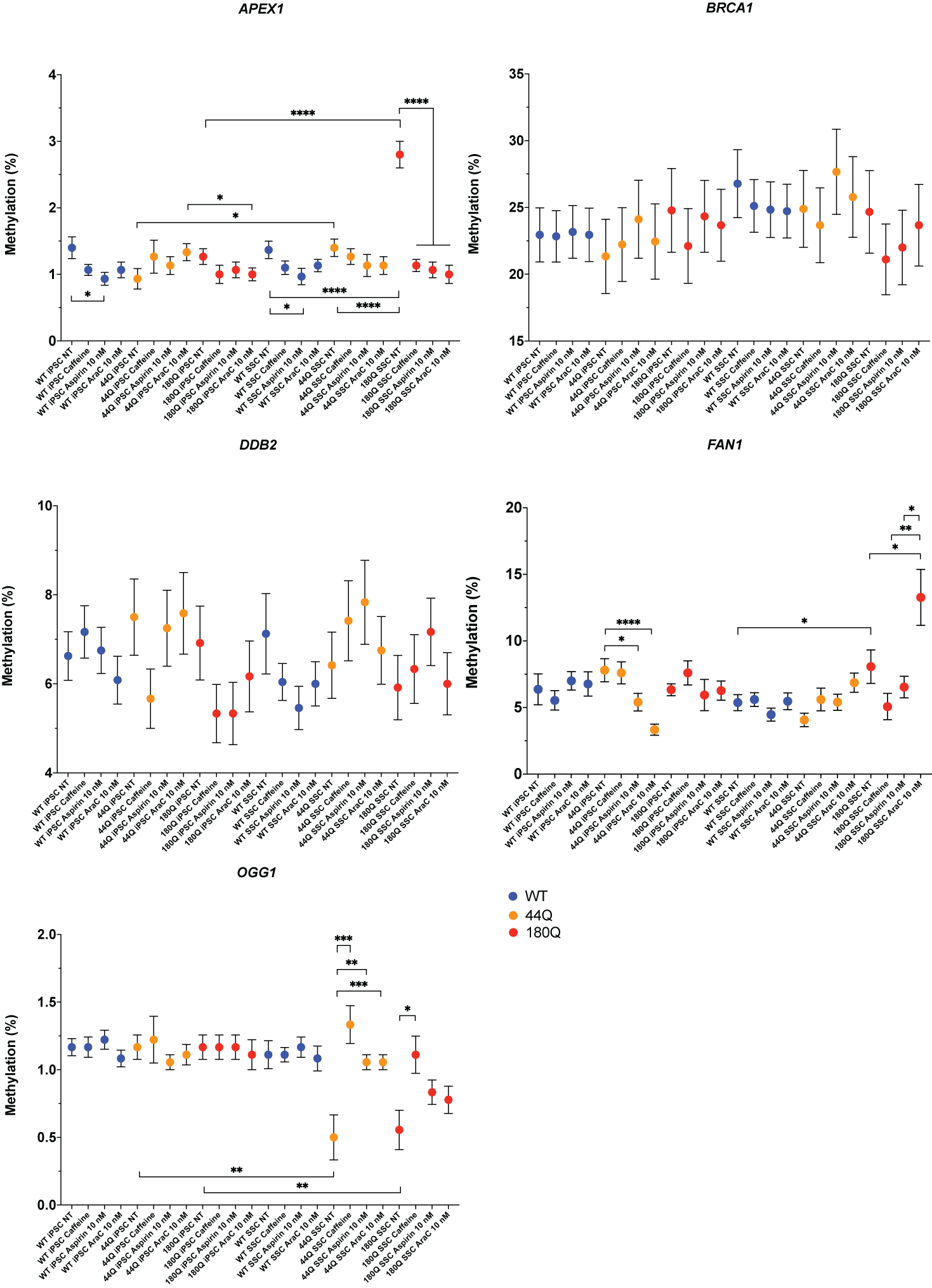

Supplement: Supplementary file 16 — (PNG 152 kb) [file 10815_2022_2594_Fig16_ESM.png]

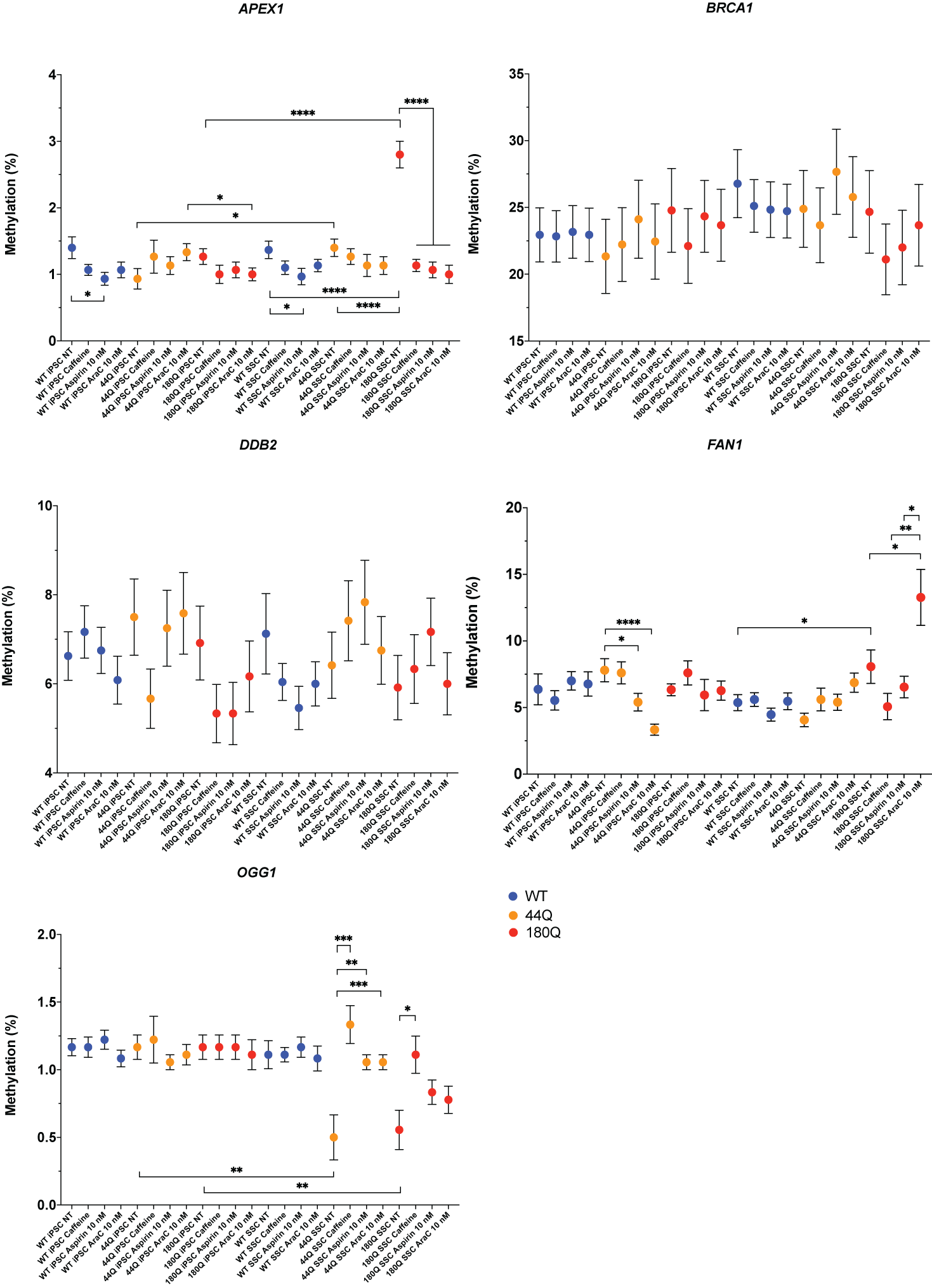

Supplement: Supplementary file 17 — High resolution image (TIF 9633 kb) [file 10815_2022_2594_MOESM9_ESM.tif]

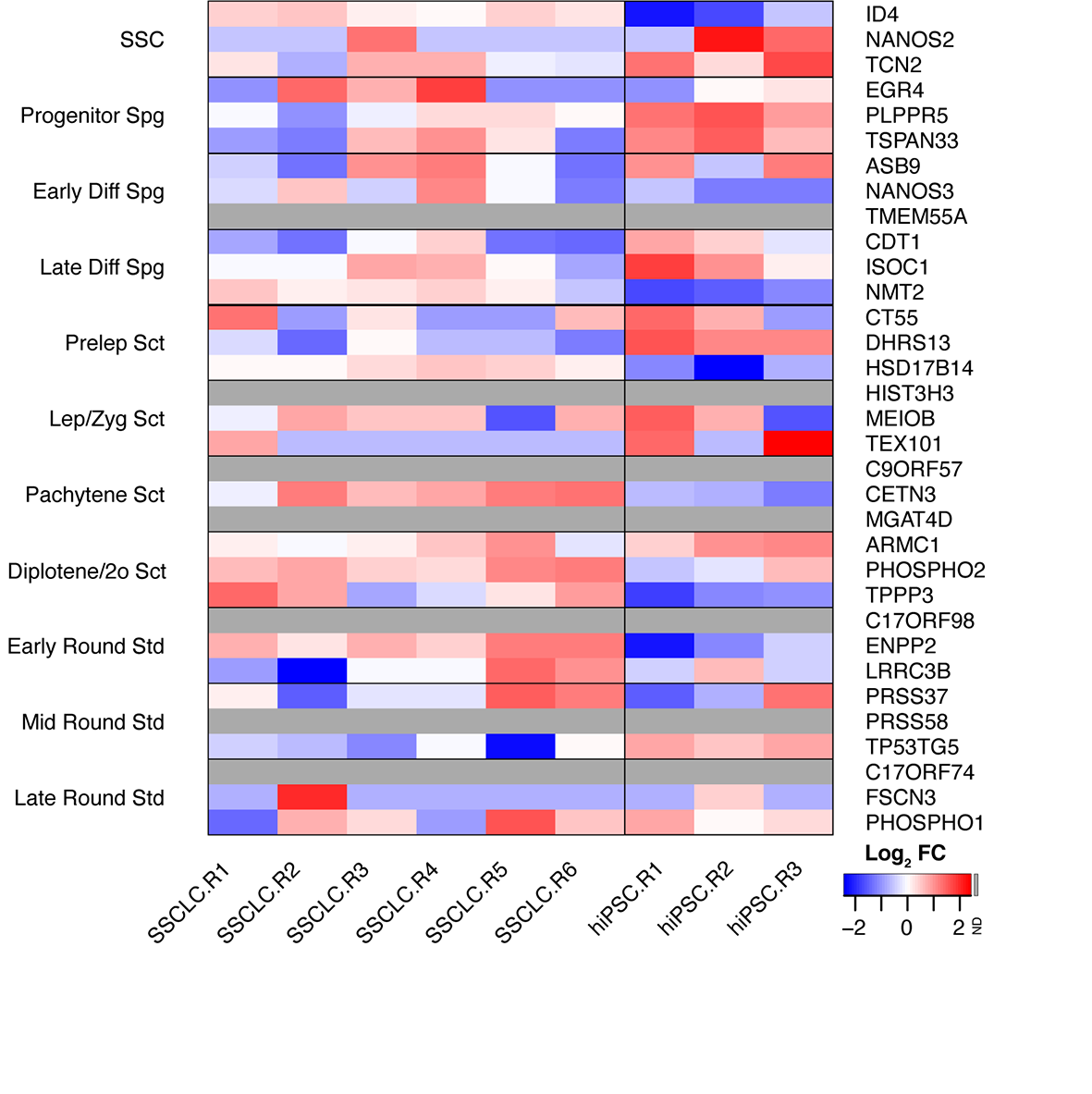

Supplement: Supplementary file 18 — (PNG 155 kb) [file 10815_2022_2594_Fig17_ESM.png]

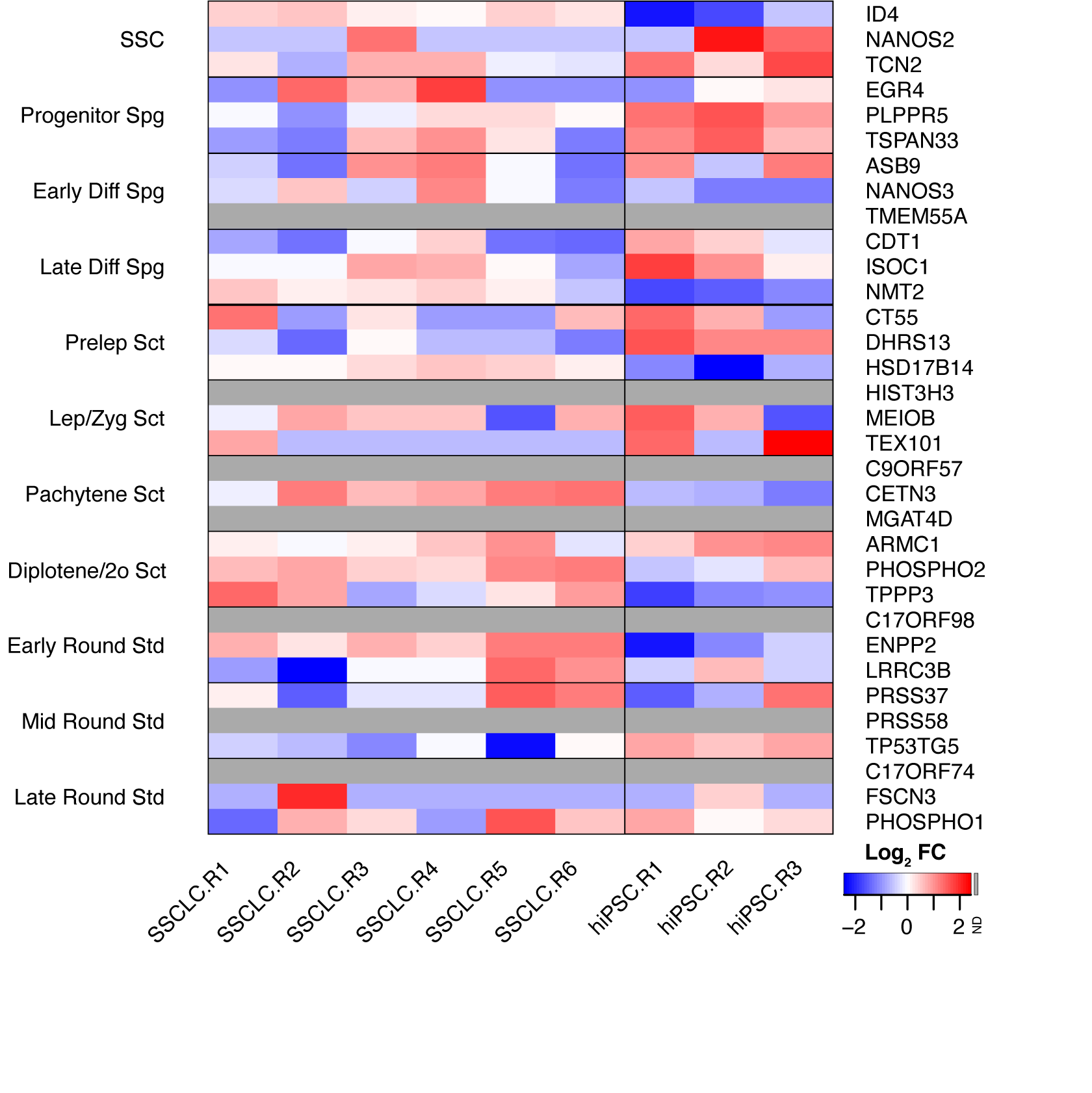

Supplement: Supplementary file 19 — High resolution image (TIF 8943 kb) [file 10815_2022_2594_MOESM10_ESM.tif]

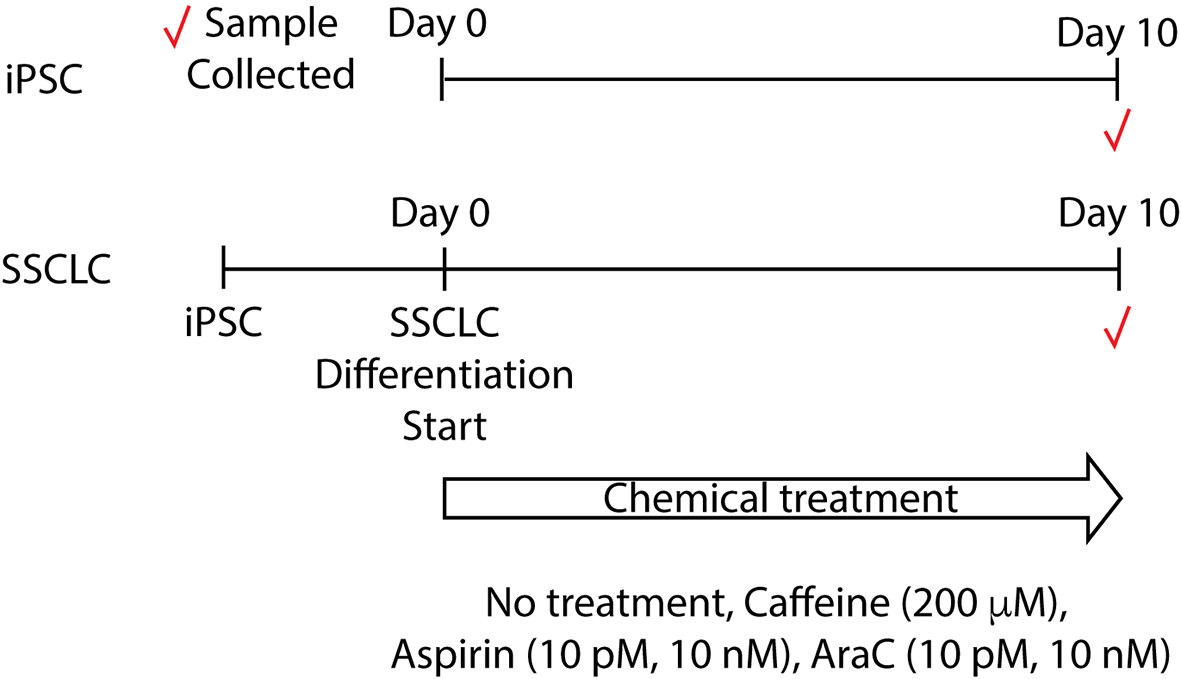

Supplement: Supplementary file 20 — (PNG 79 kb) [file 10815_2022_2594_Fig18_ESM.png]

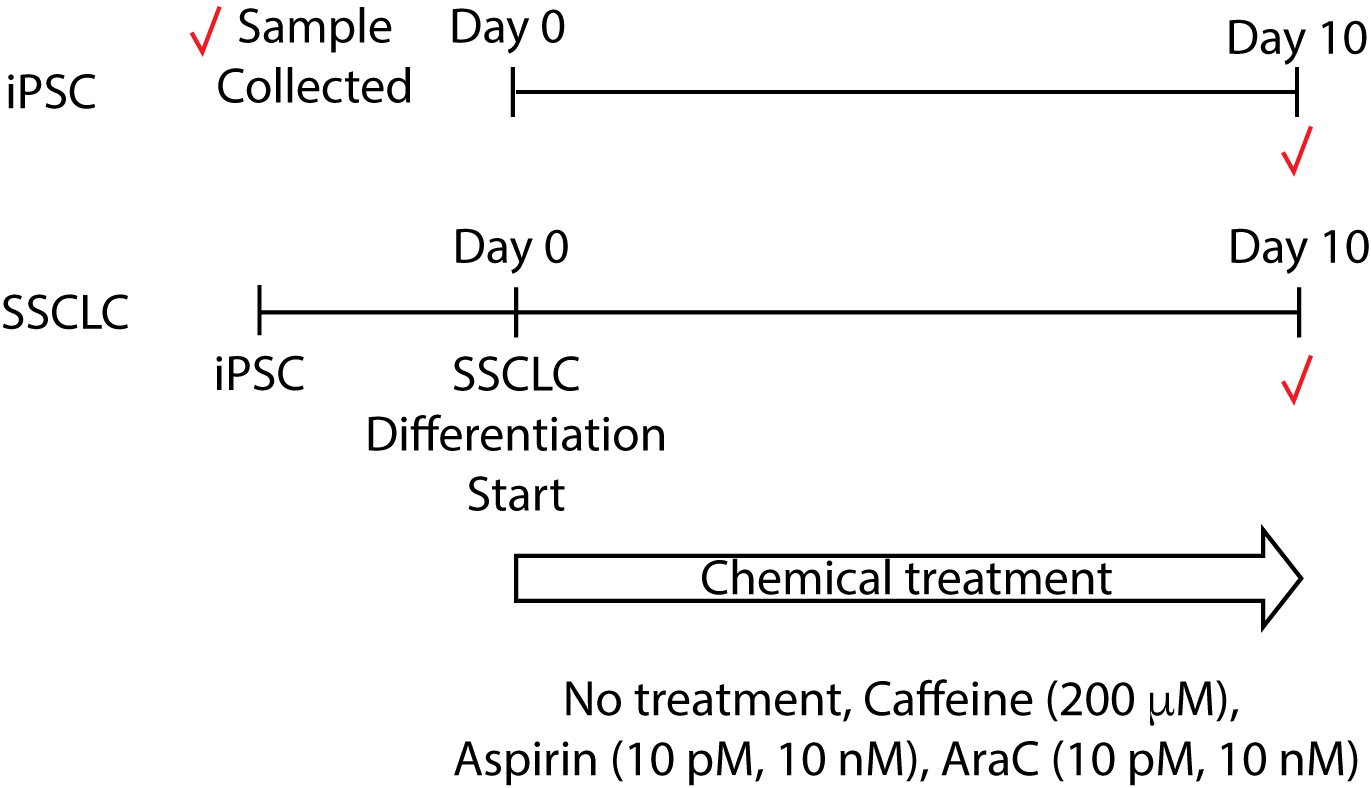

Supplement: Supplementary file 21 — High resolution image (TIF 3429 kb) [file 10815_2022_2594_MOESM11_ESM.tif]
